# Association between attendance at a behavioral change communication module and dysmenorrhea prevalence among female university students: A propensity score matched comparative study

**DOI:** 10.1371/journal.pone.0349064

**Published:** 2026-05-12

**Authors:** Liton Chandra Sen, Md Jamal Uddin, Ishrat Jahan, Nadia Salekin, Jahid Hasan Shourove, Md Mosiur Rahman, Cuilin Zhang, Davidson H. Hamer, GM Rabiul Islam

**Affiliations:** 1 Department of Community Health and Hygiene, Faculty of Nutrition and Food Science, Patuakhali Science and Technology University, Patuakhali, Bangladesh; 2 Department of Food Engineering and Tea Technology, Shahjalal University of Science and Technology, Sylhet, Bangladesh; 3 Department of Statistics, Shahjalal University of Science and Technology, Sylhet, Bangladesh; 4 Department of Population Science and Human Resource Development, University of Rajshahi, Rajshahi, Bangladesh; 5 Yong Loo Lin School of Medicine, National University of Singapore, Singapore, Singapore; 6 Department of Global Health, Boston University School of Public Health, Boston, Massachusetts, United States of America; 7 Section of Infectious Diseases, Department of Medicine, Boston University Chobanian & Avedisian School of Medicine, Boston, Massachusetts, United States of America; University of Health Sciences (Istanbul, Türkiye), TÜRKIYE

## Abstract

**Background:**

Dysmenorrhea is the most common menstrual disorder among young women and often disrupts daily activities and well-being. Although pharmacological management is widely used, sustainable non-pharmacological strategies remain underexplored, particularly in low-resource settings. This study assessed the association between a behavioral change communication (BCC) module and dysmenorrhea among female university students in Bangladesh.

**Methods:**

A matched cross-sectional comparative study initially recruited 498 female students from three public universities. Students attending three BCC sessions were classified as exposed group, while those who did not attend served as non-exposed group. After exclusions, 472 participants were analyzed. Propensity score matching (1:1 nearest-neighbor, caliper of 0.01 and no replacement) yielded 98 matched pairs. The primary measure was the average treatment effect on the treated (ATT), estimated using a doubly robust method to evaluate the association between BCC exposure and dysmenorrhea prevalence in the matched samples.

**Results:**

In matched samples, the overall prevalence of dysmenorrhea was 69.4%. Prevalence was higher among non-exposed participants (77.6%) compared with those exposed to the BCC module (61.2%). Using the doubly robust estimator, BCC-exposed participants had a 23 percentage-point lower prevalence of dysmenorrhea than non-exposed participants (ATT = −0.23; 95% CI: −0.33 to −0.13; p < 0.001), after adjustment for observed covariates. This association remained consistent and statistically significant across regression adjustment and inverse probability weighting estimators of the ATT. Participants exposed to BCC module more frequently reported regular physical activity and higher dietary diversity, both associated with lower odds of dysmenorrhea in post-matching analyses.

**Conclusion:**

Exposure to a theory-driven BCC module was associated with lower reported dysmenorrhea prevalence and healthier lifestyle behaviors among female university students in Bangladesh. Due to non-random design and lack of baseline outcome data, results are associative rather than causal, highlighting the need for further longitudinal or randomized studies to evaluate the impact of BCC programs.

## Introduction

Dysmenorrhea, characterized by painful menstrual cramps of uterine origin, is a highly prevalent yet often under-recognized gynecological disorder affecting adolescents and women of reproductive age worldwide [1]. Although common, it is frequently underdiagnosed and undertreated, as many affected women do not seek medical care, resulting in substantial physical discomfort and interference with daily activities [1,2]. Dysmenorrhea is categorized as primary dysmenorrhea (PD) and secondary dysmenorrhea. In particular, PD is diagnosed as painful menstrual cramps in the absence of any identifiable pelvic abnormalities or underlying pathological conditions [3,4]. The pain usually begins shortly before or at the onset of menstrual bleeding, lasts approximately 48–72 hours, and is most severe during the first couple few days of menstruation [5]. Usually, pain is experienced in the lower abdomen and may radiate to the lower back or upper thighs. It is often accompanied by symptoms such as dizziness, nausea, vomiting, diarrhea, fatigue, headache, insomnia, depression, and emotional instability, which can negatively impact overall quality of life (QoL) compared to healthy women [3,5,6]. A critical review by Lacovides et al. reported substantial variation in the prevalence of PD (45%–95%), largely due to inconsistencies in definition, diagnostic criteria, and assessment methods across studies [7]. In contrast, a recent systematic review and meta-analysis conducted across more than 70 countries by Arruda et al. estimated the pooled prevalence of PD to be 73% [8].

Secondary dysmenorrhea, in contrast, refers to pelvic pain resulting from underlying gynecological pathology, including conditions such as endometriosis, uterine fibroids, ovarian cysts, chronic pelvic inflammatory disease, cervical stenosis, and other structural or functional abnormalities of the reproductive organs [7,9]. Primary dysmenorrhea is primarily attributed to excessive production of prostaglandins, which induces strong uterine contractions and reduced uterine blood flow, resulting in pain [3]. The severity of symptoms may be further exacerbated by elevated levels of serum vasopressin and estradiol [10]. Moreover, several studies have reported significantly higher prostaglandin levels in women with dysmenorrhea compared with women without dysmenorrhea [11,12].

Conventional treatments for dysmenorrhea, including non-steroidal anti-inflammatory drugs (NSAIDs) and oral contraceptive pills (OCPs), are widely used due to their proven efficacy in reducing menstrual pain [13]. NSAIDs alleviate pain primarily by inhibiting cyclooxygenase enzymes (COX-1 and COX-2) and reducing prostaglandin synthesis, while OCPs suppress ovulation and thin the endometrial lining, thereby decreasing uterine contractility and menstrual pain [6,13–15]. Despite their clinical effectiveness, these medications are often associated with adverse side effects such as nausea, vomiting, gastrointestinal bleeding, acne, and asthma, particularly with prolonged use [6]. Moreover, pharmacological therapies fail to adequately relieve symptoms in approximately 20–25% of cases, highlighting the need to explore alternative approaches for dysmenorrhea management [14,16].

Behavioral approaches have been widely studied in relation to pain conditions such as osteoarthritis, cancer, and chronic pain, with several studies reporting improvements in pain related outcomes and coping mechanisms [17–19]. Emerging evidence also indicates that such behavioral and psychological strategies may be associated with reduced pain intensity and improved symptoms management among women experiencing moderate to severe dysmenorrhea [2,13,20]. While dysmenorrhea is often managed with alternative medicine and self-care practices, increasing attention has recently been directed toward psychological and behavioral approaches that modify thoughts, emotions, and behaviors to promote positive coping strategies [21,22]. Rather than directly targeting underlying organic pathology, these approaches focus on strengthening psychological resilience and physical coping capacity as non-pharmacological strategies for relieving PD-related pain [19,23].

In recent decades, lifestyle modifications through behavioral approaches and educational training programs have emerged as popular alternative strategies for managing menstrual disorders [21,24,25]. A balanced diet and regular physical activity, like yoga have been associated with improved circulation, reduced muscle tension, and potentially lower menstrual pain severity in some studies [26–28]. However, relatively few studies globally have systematically examined these lifestyle-related approaches within the context of dysmenorrhea, particularly those emphasizing broader behavioral change frameworks designed to support sustainable health behaviors [24,29].

In Bangladesh, research on non-pharmacological approaches for dysmenorrhea management remains limited. Findings from our previous study conducted in similar university settings highlighted lifestyle-related factors that may be relevant to menstrual health and suggested the potential value of behavioral change commutation (BCC) based approaches [30]. BCC, guided by the stages-of-change model, represents a structured behavioral framework that targets knowledge, attitudes, beliefs, and health related behaviors. By tailoring strategies to an individuals’ readiness to change, BCC aims to strengthen self-efficacy, facilitate informed decision-making, and promotes the adoption and maintenance of healthier lifestyle practices [31,32]. These initiatives may be particularly beneficial for young women, including university students, who are in a critical life stage for establishing long-term health behaviors. Nevertheless, empirical evidence evaluating the feasibility and potential benefits of BCC module for menstrual health outcomes remains scarce.

Therefore, this study aimed to assess the association between participation in a BCC module and the prevalence of dysmenorrhea among female university students in Bangladesh. Additionally, we examined whether students who participated in the BCC module reported differences in healthier lifestyle-related practices, including physically activity level and dietary diversity, compared with non-participants, and whether these practices were associated with variations in dysmenorrhea symptoms.

## Methodology

### Ethical approval and trial registration

The study protocol was approved by the Research Ethics Board (ref. no. AST/002/258, October 24, 2022) of Shahjalal University of Science and Technology (SUST). The procedures followed the ethical standards according to the 7^th^ revision of the Declaration of Helsinki [33]. Data were collected after obtaining written informed consent (S1 File in S1 Data) from each participant and approval from the university authorities. Privacy, anonymity, and confidentiality were ensured throughout data collection, analysis, and report writing.

The study involved no known risks, and no payments were made to participants. This study was registered on ClinicalTrials.gov (Identifier: NCT07047222) after completion of data collection, as the BCC module had been conducted prior to recognizing the requirement for prospective registration. All procedures, including participant recruitment, delivery of BCC sessions, and outcome assessment, were conducted according to a pre-specified protocol. The registration provides full transparency regarding the study protocol, outcome measures, and analysis plan. However, the authors confirm that all ongoing and related trials involving this BCC module are registered.

### Description of BCC module

The BCC module was designed to provide information on menstrual disorders, particularly dysmenorrhea, and to promote awareness of lifestyle and health-related behaviors that may influence menstrual health outcomes. The program was conceptually grounded in the Transtheoretical Model (TTM), which recognizes behavior change as gradual and progressive process [32,34]. Accordingly, the module guided participants through stages of behavioral change, beginning with awareness during the pre-contemplation and contemplation phases, followed by preparation and initiation of healthier behaviors, and concluding with strategies to reinforce and maintain these practices. awareness, preparation, initiation, and maintenance of health-related practices [34]. The educational content therefore focus on improving knowledge, enhance motivation, and strengthen self-efficacy to facilitate healthier behavioral choices.

The module covered three main thematic areas: menstrual health education, lifestyle modification, and physical activity practices. Educational components included basic menstrual physiology, common menstrual disorders such as dysmenorrhea, and both modifiable and non-modifiable risk factors including poor dietary diversity, physical inactivity, food cravings (high-fat and high-sugar foods), overweight and obesity, psychological stress, insufficient sleep, excessive caffeine intake, early age at menarche, and family history. The sessions also emphasized healthy on dietary practices, including balanced and diverse food intake, regular meals, and adequate hydration and the importance of key micronutrients relevant to women’s health such as vitamin A, vitamin B-complex, vitamin C, iron and calcium. In addition, participants were introduced to the benefits of regular physical activity for pain reduction, stress management and overall well-being. Simple stretching exercises, breathing techniques, and selected yoga postures suitable for practice in dormitory or home settings were demonstrated, while discussions addressed sleep hygiene, stress management and healthy weight maintenance as supportive lifestyle practices.

The BCC module was delivered through structured and interactive educational sessions facilitated by two trained female educators using a standardized facilitator manual, presentation slides and participant handouts. The educator had received prior training in menstrual health from a public health expert and a gynecologist to ensure appropriate delivery of the content. The sessions were held in common areas or designated meeting rooms within the residential halls to ensure a comfortable and accessible learning environment. The teaching methods included multimedia presentations, guided discussions, practical demonstration of yoga and physical activities, distributions of illustrated informational pamphlets (S1 Fig in S1 Data). Interactive activities such as quizzes and group reflections were used to reinforce learning and encourage peer engagement. Attendance was recorded for each session, and regular coordination with the research team and supervision by the principal investigator ensured fidelity to the study protocol. Participants were encouraged to set personal goals related to diet, physical activity, hydration, sleep, and caffeine intake, while reflecting on potential barriers to adopt healthier behavior. Light refreshments, small gifts, and certificates of participation were provided to encourage engagement. The detailed structure and content of the BCC module are provided in S1 Appendix in S1 Data and the corresponding logic model is shown in S2 Appendix in S1 Data.

### Study design

This study employed a matched cross-sectional comparative design to examine the association between attendance at a BCC module and dysmenorrhea prevalence among female university students. Random assignment to groups was not feasible due to fixed class schedules, residential hall arrangements, academic commitments, and voluntary participation in BCC sessions. Therefore, participants were classified based on their exposure to the BCC module. Students who voluntarily enrolled between May 1 and May 15, 2023 and completed all three BCC sessions were designated as the BCC-exposed group. The comparison group consisted of female students from the same universities who did not participate in any component of the BCC module during the study period, referred to as the BCC non-exposed (control) group. Control participants were identified from official dormitory and student enrollment records to ensure comparability in terms of academic environment, living conditions, and access to university health services. They were not approached to attend at the sessions and did not receive any BCC-related materials or follow-up communication during the study period.

The BCC sessions were conducted between May 20 and June 30, 2023, at three public universities in Bangladesh: Patuakhali Science and Technology University (PSTU), Barisal University (BU), and Khulna University (KU). These universities were purposively selected to ensure geographic diversity and to represent female students from coastal and southern regions of the country, where access to menstrual health education and support services is often limited. Moreover, these institutions have a high number of female students residing in dormitories, which facilitated participant recruitment and program delivery. The sessions were delivered in small peer groups of approximately 20 participants, with each group attending three sessions, one of each domain of the BCC module. A total of 39 sessions were conducted, with two sessions delivered per day. Each session lasted 60–90 minutes and followed a standard structure to ensure consistency across groups and study locations.

Several measures were taken to minimize contamination between the study groups. BCC sessions were conducted in designated residential hall spaces with restricted access and scheduled separately from the routine academic activities. Attendance was strictly monitored, and educational materials (including printed pamphlet and visual aids illustrating yoga postures) were distributed exclusively to participating students. The participants were also requested not to share the training content with other students during the study period.

Following the completion of BCC sessions, participants in the BCC-exposed group were contacted periodically over a six-month follow-up period (July 15, 2023 to January 15, 2024). During this time, the educators, under the supervision of a research supervisor, conducted reinforcement visits at the second, fourth, and six months. These visits provided clarification on session content, addressed participants’ questions, and facilitated engagement with the practices discussed in the BCC sessions. The follow-up period was intended to support participants in applying the information covered in the three content domains of the BCC module and to enable descriptive monitoring of participant experiences, without implying causal effects on behavior or health outcomes.

Finally, the outcome assessment for both the BCC-exposed and matched control groups was conducted from February 1, 2024 to March 31, 2024. Data were collected at the individual level, allowing for a comparative evaluation of the association between BCC exposure and menstrual health-related outcomes, including dysmenorrhea prevalence.

### Participants

#### Eligibility criteria.

The study population consisted of full-time female undergraduate students aged 19–25 years. Age eligibility was based on self-report and verified through university enrollment records. This age range was selected to capture young adult women most likely to experience menstrual disorders. Eligible participants were required to be willing to attend all phases of the study, including three scheduled sessions of the BCC module. Participants were excluded if they were currently pregnant or breastfeeding, as confirmed during the initial screening, to reduce hormonal variability and ensure comparability across participants.

#### Recruitment methods and settings.

Participants were recruited from a total population of 5,298 female students enrolled at the three public universities (PSTU, BU, and KU). Recruitment was conducted through a combination of in-person briefings, dormitory announcements, and university notices posted within the residential halls, and allowing students to voluntarily enroll in the BCC sessions. Trained female research assistants led the recruitment process by providing detailed information about the study and conducting initial eligibility screening. A purposive sampling strategy was used to ensure that participants in both groups met the inclusion criteria. All recruitment activities were conducted inside the university dormitories to ensure accessibility and to engage students in a familiar, comfortable environment. Eligible students who agreed to participate provided informed consent prior to enrollment.

### Sample size estimation

The sample size for this study was calculated based on the anticipated prevalence of dysmenorrhea in the non-exposed group, which was 42% as reported in our previous study [30]. A 1:1 proportional sampling strategy was applied to ensure an equal number of participants in the BCC-exposed and non-exposed (control) group, maintaining a balanced comparison between the study arms. To detect a difference of 30% in dysmenorrhea prevalence between the BCC-exposed and non-exposed groups, a total of 452 participants were required, with 226 participants in each group. [35,36]. The proportional distribution of the sample included 56, 119 and 51 respondents for each independent group from PSTU, BU, and KU, respectively. The sample size was calculated based on a 95% confidence interval (CI) and 80% statistical power using the following equation (see S1 Text in S1 Data for detail of the sample size calculation):


$$\text{n}=\frac{{\left(Z_{\propto /\text{2}\;}+Z_{\beta }\right)}^{\text{2}}\;\times \;\left[P_{\text{1}\;}\left(\text{1}-P_{\text{1}\;}\right)\;+P_{\text{2}\;}\left(\text{1}-P_{\text{2}\;}\right)\;\right]\;}{{\left(P_{\text{1}\;}-\;P_{\text{2}\;}\right)}^{\text{2}}}$$

Whereas, n = required sample size per group

$P_{\text{1}\;}=\;\text{0}.\text{42}$ (42% anticipated incidence of outcome in the non-exposed group)

$P_{\;\text{2}}=\;\text{0}.\text{294 }$ (expected 30% relative difference from 42% in the BCC-exposed group)

$Z_{\propto /\text{2}\;}=\text{1}.\text{96 }$ (Z value for 95% confidence interval)

$Z_{\beta }=\;\text{0}.\text{84 }$ (Z value for 80% power)

$P_{\text{1}\;}\left(\text{1}-P_{\text{1}\;}\right)\;=\;\text{0}.\text{42 }\times (\text{1}\;-\;\text{0}.\text{42})\;=\;\text{0}.\text{25 }$ (estimated variance for non-exposed group)

$P_{\text{2}\;}\left(\text{1}-P_{\text{2}\;}\right)\;=\;\text{0}.\text{294 }\times \;(\text{1}\;-\;\text{0}.\text{294})\;=\;\text{0}.\text{21 }$ (estimated variance for BCC-exposed group)

### Participants flow

**Fig 1:**
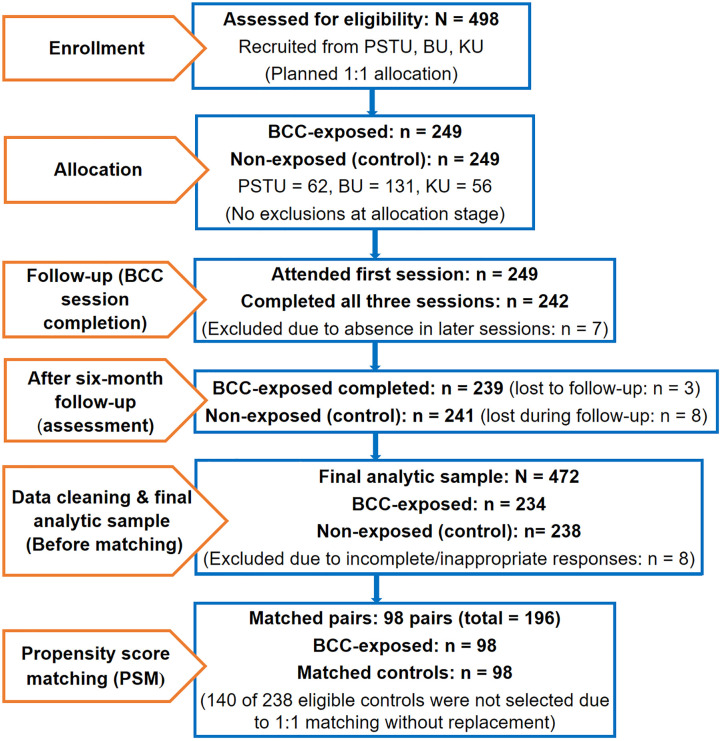
TREND flowchart of sampling process.

Initially 498 participants were recruited from three public universities (PSTU, BU, and KU), evenly divided between the BCC-exposed and non-exposed groups (249 participants per group), accounting for an anticipated 10% non-response rate. After explaining the purpose of the study and obtaining informed consent, 249 students (62 from PSTU, 131 from BU, and 56 from KU) were enrolled in the BCC-exposed group. An equal number of students from the same universities were allocated to the non-exposed comparison group. Although all listed participants attended the first session, seven students (two, four, and one from PSTU, BU, and KU, respectively) were excluded due to absence during the second and third sessions of the BCC module. Consequently, the remaining 242 participants who completed all three BCC sessions were included in the follow-up phase. During the six-month follow-up, a total of 11 participants (3 BCC-exposed and 8 non-exposed) declined to participate in the outcome assessment. Therefore, 239 BCC-exposed and 241 non-exposed students completed the final outcome assessment. Data from eight participants were subsequently excluded due to incomplete or invalid responses, resulting in a final analytic sample of 234 BCC-exposed and 238 non-exposed participants. Fig 1 demonstrates the entire sampling process and participation flow.

### Data collection

The data were collected on-site at the respective universities, in private rooms within dormitories or academic buildings to maintain confidentiality and minimize distractions. Within these settings, a standardized data collection approach was followed to ensure consistency across study sites. Data were gathered using structured questionnaires specifically designed for the context of Bangladesh, informed by a comprehensive review of relevant literature and information, education, and communication (IEC) materials on menstrual disorder management [25,30,37,38]. The questionnaires were initially developed in English and then translated into Bengali (S2 File in S1 Data). A reverse translation process was also conducted to verify the accuracy and maintain consistency with the original questionnaire. Data were collected using these questionnaires administered to respondents in both the BCC-exposed and non-exposed groups. A team comprising two supervisors and ten data collectors was recruited and trained in social science research methods, public health management, ethical principles, data collection tools, interviewing techniques, and the verbal multidimensional scoring system used to classify outcomes. Recognizing the sensitive nature of the topic, all data collectors and supervisors were female to create a comfortable environment for the participants to discuss menstrual-related issues. However, blinding of outcome assessors to the group allocation was not implemented during data collection. Before the actual data collection began, a pilot study was conducted with 45 participants to assess the validity, reliability and feasibility of the designed questionnaires.

### Outcome variable

Dysmenorrhea was defined as any pain or discomfort associated with the menstrual cycle and assessed according to the scoring system of Andersch and Milsom [39]. This system categorized dysmenorrhea into four grades: grade 0, grade 1, grade 2, and grade 3, based on the severity of pain, interference with daily activities, and the dependence on analgesic use. For the purpose of the statistical analysis, dysmenorrhea was treated as a binary outcome variable. Respondents reporting no pain were categorized as “No = 0”, while those experiencing any level of pain (mild, moderate, or severe) were grouped as “Yes = 1” [40,41]. Dysmenorrhea severity was assessed using a standardized, interviewer-administered grading scale. All interviewers underwent comprehensive training on the scoring criteria and followed a structured, step-by-step assessment protocol covering all components of symptom severity before assigning the final grade. Before data collection, interviewers read the instructions to participants and clarified any unclear terms to ensure consistent understanding. This approach was used to minimize misclassification and enhance the reliability of the self-reported outcome measures. The detailed description of the outcome variable is provided in S2 Text in S1 Data.

### Covariates

Appropriate selection of covariates is essential in PSM to improve comparability between exposure groups and reduce potential confounding. Covariates were selected based on prior literature, theoretical relevance, and their potential association with both BCC exposure and dysmenorrhea outcomes [42]. Physical activity was categorized as follows: i) sedentary (referring light-intensity physical activity, e.g., casual walking, light household chores; and activities with little or no exercise); (ii) active, (moderate-intensity activities such as brisk walking, light jogging, or moderate exercise, e.g., yoga, weight lifting etc. for at least 150–300 minutes per week); and (iii) athlete (vigorous-intensity activities such as running, jumping, swimming, climbing, fast cycling or competitive sports etc. for at least 75–150 minutes per week) based on World Health Organization’s (WHO) recommendations [43]. Obesity was assessed using body mass index (BMI, kg/m^2^) as a nutritional indicator. We classified BMI based on the WHO-recommended cut-off points for the Asian population [44], as follows: (i) underweight (< 18.5 kg/m²), (ii) normal weight (18.5–22.9 kg/m²), (iii) overweight (23.0–27.5 kg/m²), and (iv) obese (> 27.5 kg/m²). However, the overweight and obese categories were combined into an overweight/obese group to avoid zero cell counts in chi-square estimating. Dietary data were collected using a 24-hour recall method over five consecutive days, and dietary diversity score (DDS) was calculated as per Food and Agriculture Organization’s (FAO) guideline [45]. The average DDS (range: 0–10) was calculated by summing the five daily scores and dividing by five. Participants were then categorized as having high DDS (≥5) or low DDS (<5) based on their average score.

We extracted data on various lifestyle factors including cravings for high-fat and sweet foods (yes/no) [46], skipping breakfast one or more times in the last week (yes/no), sleep duration (< 7 hours per night, ≥ 7 hours per night), and caffeine consumption (infrequent, < 3 times per week; frequent, ≥ 3 times per week) [37]. Additional socio-demographic and nutritional covariates included family history of menstrual disorders (yes/no), age at menarche (years), and marital status (never married, ever married), father’s and mother’s education (below secondary education, secondary or higher education), and mother’s occupation (formal occupation, informal occupation). A comprehensive description of each covariate used in this study is presented in S3 Text in S1 Data.

### Statistical analysis

Following data collection, propensity score matching (PSM) was applied to improve comparability between the BCC-exposed and non-exposed groups. The PSM approach estimates the conditional probability of exposure to the BCC module given a set of observed covariates [47] and is widely used in observational studies where random assignment is not feasible [48,49], helping to reduce potential confounding due to measured variables [50].

To implement this approach in the present study, propensity scores (PS) were first estimated for each participant using a logistic regression model with all the observed covariates implemented through the “psmatch2” command. Seven candidate propensity score models (Model 1–7), each incorporating different combinations of theoretically relevant covariates, were evaluated. The selection of candidate covariates was informed by prior literature and subject-matter knowledge rather than statistical significance of outcome. These alternate specifications were examined to assess the stability of covariate balance among the models.

Across all candidate models, the primary matching algorithm was held constant and implemented as 1:1 nearest-neighbor matching without replacement and a caliper width of 0.01 [51,52]. This approach paired each BCC-exposed participant with a non-exposed participant who had the closest propensity score within the specified caliper. Simulation studies indicate that tighter calipers reduce residual bias and improve match precision, while maintaining appropriate confidence interval coverage and type I error rates, particularly for binary outcomes and when covariates are binary or mixed in distribution [53,54]. Therefore, among the non-exposed participants, 238 fell within the common support region, indicating sufficient overlap in the propensity score distributions between the exposure groups. Applying the specified matching criteria resulted in 196 matched samples (98 BCC-exposed and 98 non-exposed participants), smaller than the initially planned sample size (Fig 1). The remaining 140 non-exposed participants, although within the common support region, were not selected due to the 1:1 matching procedure and the no-replacement rule, reflecting constraints of the matching algorithm rather than inadequate overlap between groups (see propensity score distribution in S2 Fig in S1 Data). This smaller matched sample was a deliberate consequence of applying strict matching criteria to ensure optimal covariate balance and strengthen internal validity. Although wider calipers could have increased the number of matched participants, they would have involved a trade-off between sample size and match precision. Given the smaller matched sample obtained through primary matching specification, additional sensitivity analyses were planned to examine the robustness of the results under alternative matching procedures. These analyses included nearest-neighbor matching with multiple neighbors, radius matching with wider calipers and kernel matching. Evaluating these alternative approaches ensured that the robustness of the findings could be assessed despite the reduced sample size in the primary matched dataset. Accordingly, a caliper width of 0.01 was selected for the primary analysis.

Following the matching procedure, covariate balance between BCC-exposed and non-exposed groups was carefully assessed. Balancing diagnostics included standardized mean differences (percentage bias), percentage reduction in bias, post-matching t-tests, and variance ratios (S1A Table in S1 Data) using the “pstest” command. Overall balance was summarized using Rubin’s B and Rubin’s R statistic (S1B Table in S1 Data). To enhance transparency and minimize the risk of specification searching, the criteria for selecting the propensity score model were defined a priori. Adequate balance was defined a priori as standardized bias within ±10% for all covariates [55,56], Rubin’s overall imbalance statistic (B) < 25% [57], and Rubin’s variance ratios (R) within the range of 0.5 to 2.0 [58]. Models were compared solely on the basis of these predefined balance diagnostics rather than outcome estimates or statistical significance, thereby reducing the risk of specification searching. The model meeting all pre-specified thresholds and demonstrating the most consistent reduction in residual imbalance across covariates was retained as primary propensity score specification.

After identifying the primary PSM model, the same set of covariates included in that model was used for adjustment in both pre- and post-matching analyses. Subsequently, the matched sample generated from the primary propensity score specification was used for post-matching outcome analysis. Before matching, bivariate and multivariable logistic regression models were fitted to examine crude and adjusted associations between BCC exposure and dysmenorrhea. After matching, conditional logistic regression (fixed-effect) was applied to the matched pairs to account for the matched study design using the “clogit” command. Bootstrap resampling was used to estimate the standard error (SE) and 95% confidence intervals for the propensity score adjusted estimates. A total of 500 bootstrap replicates were performed, which provided stable SEs and CIs, as additional replicates had minimal impact on their precision and coverage [59].

The categorical variables were expressed as numbers and percentages and compared using chi-square (𝛘^2^) test while the continuous variables were presented as means and standard deviations (SD) and compared using Student’s t-test. Crude odds ratios (COR) and adjusted odds ratios (AOR) are reported with corresponding confidence intervals. Analyses were conducted using Stata 17.0, with statistical significance defined at a two-sided α = 0.05. Significance levels were indicated as * *p* < 0.05, ** *p* < 0.01, and *** *p* < 0.001. Although the BCC module was delivered in group sessions, the individual participants were considered the unit of analysis.

### Sensitivity analysis

#### Assessing alternative cut-points for dichotomization of dysmenorrhea severity.

To examine whether the findings were robust when the full ordinal structure of dysmenorrhea severity was retained, an ordered logistic regression model was conducted using the original four-grade pain scale (no pain, mild, moderate, and severe). The model was fitted to the unmatched sample and adjusted for the same covariates included in the primary multivariable regression analysis. This analysis evaluated whether the direction and magnitude of associations were consistent when the full ordinal outcome was considered.

#### Robustness to matching algorithms.

Sensitivity analyses were conducted to assess the robustness of the observed association between BCC exposure and dysmenorrhea across alternative matching specifications relative to the primary 1:1 nearest-neighbor matching algorithm. These specifications included nearest-neighbor matching with two and three neighbors, radius matching with calipers of 0.01, 0.05, and 0.10 and kernel matching. All matching procedures used the same set of observed covariates. Matching was restricted to the region of common support to ensure adequate overlap between BCC-exposed and non-exposed participants. Using the propensity score-matched sample, the ATT (average treatment effect on the treated) estimate represents the average difference in dysmenorrhea prevalence between BCC-exposed and non-exposed counterparts. The standard errors (SEs) and 95% CIs were obtained using the same bootstrap procedure with 500 replications as described earlier. Consistency in the magnitude and direction of the estimates across matching algorithms was used to assess the robustness of the findings.

#### Robustness to estimation approaches.

To further assess robustness, alternative propensity score based estimation approaches were applied, including regression adjustment (RA), inverse probability weighting (IPW), and inverse probability weighting with regression adjustment (IPWRA). These approaches provide model based estimation of adjusted differences in dysmenorrhea prevalence between BCC-exposed and non-exposed participants while accounting for observed covariates. IPWRA was applied as doubly robust estimation approach which combines outcome regression and propensity score weighting within a single framework. This approach has the advantage of providing stable estimates when either exposure model or the outcome model is correctly specified, thereby improving robustness to model misspecification. Robust SE were used to construct 95% confidence intervals. ATT estimates were reported as the primary measure, while average treatment effect (ATE), representing the average differences in dysmenorrhea prevalence across the overall population under the model specification, was also estimated for comparison. Both ATT and ATE were obtained using “teffects” command (see S4 Text in S1 Data for detailed specification). Covariate balance between exposure groups after matching was verified prior to outcome estimation.

#### Estimation of log bayes factor (LBF).

Bayesian logistic regression was applied to complement the frequentist analysis and further to assess the robustness of the observed associations between BCC module attendance and dysmenorrhea. This approach allows the strength of evidence to be quantified while accounting for model uncertainty. Posterior means of the coefficients were reported as odds ratios (ORs) with 95% credible intervals (CrIs). Weakly informative normal priors were specified for all regression coefficients (mean = 0, SD = 2.5). A Bernoulli likelihood with a logit link function was assumed for the binary outcome. Posterior distributions were approximated using Markov Chain Monte Carlo (MCMC) sampling with 40,000 total iterations with 5000 burn-in iterations and a thinning interval of 5 (see S5 Text in S1 Data for details). For both unmatched and matched samples, LBFs were calculated as the difference between the log marginal likelihood of the alternative model (including BCC attendance) and the null model (excluding BCC attendance). An LBF value greater than 10 was interpreted as strong evidence in favor of the model including BCC attendance, according to conventional Bayesian model comparison criteria [60].

#### Impact of potential contamination on the observed association.

Despite the preventive measures, participants from both groups resided within the same university setting, allowing the possibility of informal peer interactions. Such interactions may have resulted in indirect exposure to BCC-related information among some control participants. To evaluate the potential influence of contamination on the observed association, a sensitivity analysis was performed assuming varying levels of indirect exposure among the control group. Corrected odds ratios (ORs) were estimated for different assumed contamination proportions (e.g., 10%, 20%, 30%, 40%, 50%) using the adjusted ORs obtained from the multivariable logistic regression model. This analysis allowed assessment of how the magnitude of the observed association might change under different contamination scenarios. The detailed method of estimation is provided in S6 Text in S1 Data.

## Results

A total of 472 participants were included in the analysis before matching, comprising 234 BCC-exposed and 238 non-exposed students. These participants represented the final sample after excluding incomplete or invalid responses. Propensity score matching yielded 98 matched pairs (196 participants) for analysis of the association between BCC attendance and dysmenorrhea.

### Selection of primary propensity score specification

**Fig 2:**
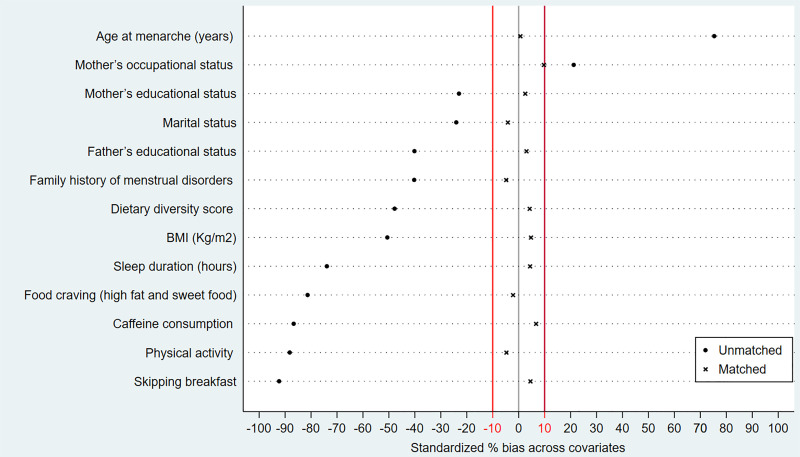
Standardized bias plot of the covariates before and after propensity score matching. The X-axis displays standardized percentage bias for each covariate, while the Y-axis lists the observed covariates. The standardized bias plot was used to assess covariate balance between the BCC-exposed and non-exposed groups before and after matching. The vertical reference line (red) on X-axis at ±10% standardized bias represents the accepted threshold for adequate covariate balance..

Among seven pre-specified propensity score specifications, only Model 2 satisfied all predefined balance criteria and was therefore retained as the primary propensity score specification (S1B Table in S1 Data). Under this model, all thirteen covariates achieved standardized bias within the ± 10% threshold, with a mean post-matching bias of 4.3%. Rubin’s B statistic (17.3%) and Rubin’s R (0.80) were also within the acceptable limits, indicating adequate overall balance. These balance metrics are further illustrated visually in Fig 2, which shows that propensity score matching substantially reduced covariate imbalances, bringing all standardized biases within the predefined ±10% range, representing improved comparability between the BCC-exposed and non-exposed groups.

**Fig 3:**
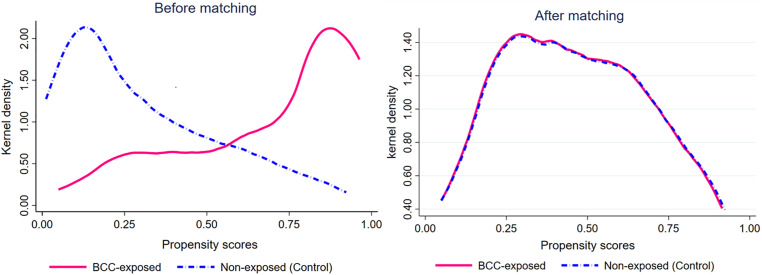
Kernel density plots of propensity scores before and after matching. X-axis: Propensity scores (range 0–1.0), and Y-axis: Kernel density (probability density of propensity scores). **(A)** Before matching: shows limited overlap between BCC-exposed and non-exposed (control) groups, **(B)** After matching: exhibits improved overlap in propensity scores, indicating better balance between groups.

In comparison, the remaining specifications did not fully satisfy the predefined thresholds. Specifically, several models exhibited residual standardized bias exceeding ±10% for one or more covariates, while others showed less favorable overall imbalance statistics, including Rubin’s R values approaching or exceeding 25% or variance ratios outside the acceptable 0.5–2.0 range (S1B Table in S1 Data). Additionally, Fig 3 demonstrates that after matching, the propensity score distributions of the BCC-exposed and non-exposed groups exhibit substantially greater overlap compared to before matching. This improved alignment further confirms enhanced comparability between groups and supports Model 2 as the primary propensity score specification for subsequent analysis.

### Participant characteristics

**Fig 4:**
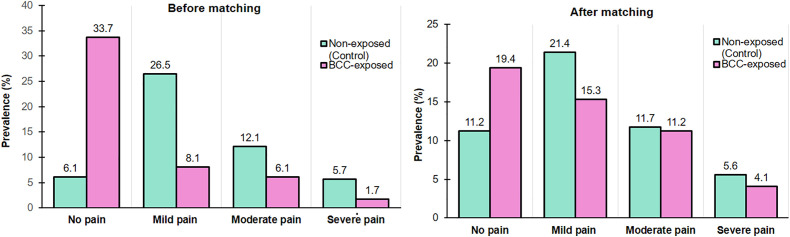
Distribution of dysmenorrhea pain grades by BCC exposure before and after propensity score matching. Prevalence (%): percentage of participants reporting different dysmenorrhea pain grades (No pain, Mild, Moderate, and Severe) in BCC-exposed and non-exposed (control groups). **(A)** Shows the distribution of dysmenorrhea pain grades in the study groups before matching (𝛘^2^ = 168.01 and p < 0.001); **(B)** Demonstrates the distribution of dysmenorrhea pain grades in BCC-exposed and non-exposed (control) groups after matching (𝛘^2^ = 6.8 and p > 0.05).

In this study, Fig 4 demonstrates the distribution of dysmenorrhea pain grades according to BCC exposure status before and after propensity score matching. Before matching, participants in the BCC-exposed group had a higher proportion reporting no pain (33.7% vs. 6.1%), whereas lower proportions reported mild (8.05% vs. 26.5%), moderate (6.1% vs. 12.08%), and severe pain (1.7% vs. 5.7%) compared with the non-exposed (control) group. These differences in pain severity distribution between groups were statistically significant (*p* < 0.001). In contrast, in the matched sample, the overall prevalence of dysmenorrhea was 69.4%, including 77.6% among non-exposed participants and 61.2% among BCC-exposed participants. Specifically, no pain was reported by 38.8% of BCC-exposed and 22.4% of non-exposed participants; mild pain by 30.6% vs. 42.9%; moderate pain by 22.4% vs. 23.5%; and severe pain by 8.2% vs. 11.2%. Although the BCC-exposed group had a higher proportion with no pain and the non-exposed group had higher pain levels, the differences in distribution were no longer statistically significant (*p* > 0.05).

Table 1 presents the characteristics of the study participants according to BCC exposure status before and after PSM analysis. Before matching, several participant characteristics differed between the BCC-exposed and non-exposed groups. Compared with the non-exposed group, participants in the BCC-exposed group were more frequently physically active or athletes (25.4% vs. 6.6%, *p* < 0.001), more likely to have normal BMI (36.2% vs. 17.6%, *p* < 0.001), higher dietary diversity (≥ 5 DDS: 29.2% vs. 18.01%, *p* < 0.001), and reported older age at menarche (13.4 ± 1.7 vs. 12.1 ± 1.5 years, *p* < 0.001). Significant differences were also observed for food craving, skipping breakfast, sleep duration, caffeine consumption, family history of menstrual disorders and father’s education (*p* < 0.001), as well as marital status, mother’s education and mother’s occupational status (*p* < 0.05), indicating imbalance in observed covariates between the groups. Following matching, the distribution of these characteristics was well balanced between the groups, with no statistically significant differences observed (all *p* > 0.05). This indicates improved balance in observed covariates in the matched sample used for subsequent analyses.

**Table 1:** Participant characteristics according to BCC-exposure status before and after propensity score matching.

Variables	Before propensity score matched			After propensity score matched		
	Non-exposed (Control) (N = 238) n (%)	BCC-exposed (N = 234) n (%)	*p*-value	Non-exposed (Control) (N = 98) n (%)	BCC-exposed (N = 98) n (%)	*p*-value
***Physical activity***						
Sedentary	206 (43.6)	114 (24.2)	< 0.001	77 (39.8)	75 (38.3)	0.732
Active and Athlete	32 (6.6)	120 (25.4)		21 (10.7)	23 (11.7)	
***BMI (kg/m*** ^***2***^ **)**						
Normal weight (18.5–22.9)	83 (17.6)	171 (36.2)		45 (23.0)	56 (28.8)	
Underweight (< 18.5)	44 (9.3)	37 (7.8)	< 0.001	25 (12.8)	18 (9.2)	0.266
Overweight/obese (> 22.9)	111 (23.5)	26 (5.5)		28 (14.3)	24 (12.3)	
***Dietary diversity score (DDS)***						
< 5 (Low)	153 (32.4)	96 (20.3)		52 (26.5)	54 (27.6)	
≥ 5 (High)	85 (18.01)	138 (29.2)	< 0.001	46 (23.5)	44 (22.5)	0.774
***Food craving (high fat and sweet food)***						
No	77 (16.3)	164 (34.8)	< 0.001	45 (23.0)	46 (23.5)	0.886
Yes	161 (34.1)	70 (14.8)		53 (27.0)	52 (26.5)	
***Skipping breakfast***						
No	72 (15.5)	169 (35.8)	< 0.001	48 (24.5)	46 (23.5)	0.775
Yes	166 (35.2)	65 (13.8)		50 (25.5)	52 (26.5)	
***Sleep duration (hours)***						
≥ 7 hours per night	75 (15.9)	155 (32.8)	< 0.001	44 (22.5)	42 (21.5)	0.773
< 7 hours per night	163 (34.5)	79 (16.7)		54 27.6)	56 (28.6)	
***Caffeine consumption***						
Infrequent (< 3 times per week)	65 (13.8)	157 (33.3)	< 0.001	42 (21.4)	39 (19.9)	0.663
Frequent (≥ 3 times per week)	173 (36.7)	77 (16.3)		56 (28.6)	59 (30.1)	
***Family history of menstrual disorders***						
No	160 (33.9)	197 (41.7)	< 0.001	69 (35.2)	71 (36.2)	0.752
Yes	78 (16.5)	37 (7.8)		29 (14.8)	27 (13.8)	
***Age at menarche (years)***						
Mean (SD)	12.1 (1.5)	13.4 (1.7)	<0.001^a^	12.6 (1.6)	12.6 (1.7)	0.996^a^
***Marital status***						
Never Married	216 (45.8)	226 (47.9)		93 (47.5)	94 (48.0)	
Ever married	22 (4.7)	8 (1.7)	0.010	5 (2.6)	4 (2.0)	0.733
***Father’s educational status***						
Secondary/Higher (> 5 y schooling)	188 (39.9)	217 (46.0)		87 (44.4)	86 (43.9)	
Below secondary (0–5 y schooling)	50 (10.6)	17 (3.6)	< 0.001	11 (5.6)	12 (6.1)	0.824
***Mother’s educational status***						
Secondary/Higher (> 5 y schooling)	176 (37.3)	195 (41.3)		78 (39.8)	77 (39.2)	
Below secondary (0–5 y schooling)	62 (13.1)	39 (8.3)	< 0.05	20 (10.2)	21 (10.7)	0.861
***Mother’s occupational status***						
Formal occupation	66 (14.0)	44 (9.3)	< 0.05	28 (14.3)	24 (12.2)	0.520
Informal occupation	172 (36.4)	190 (40.3)		70 (35.7)	74 (37.8)	

^a^According to the t-test and other p-values are based on chi square test. BMI = body mass index; SD = standard deviation.

### Determinants of dysmenorrhea

Table 2 presents the association between lifestyle and dietary factors and the likelihood of experiencing dysmenorrhea according to BCC-exposure status, based on multivariable logistic regression analyses conducted before and after matching. Compared with sedentary participants, being physically active among BCC-exposed participants was associated with 77% and 94% lower odds of dysmenorrhea before matching (AOR = 0.23; 95% CI 0.07, 0.68; *p* < 0.01) and after matching (AOR = 0.06; 95% CI 0.03, 0.40; *p* < 0.01). Similarly, a high dietary diversity score (DDS ≥ 5) was associated with a substantially lower likelihood of dysmenorrhea among BCC-exposed participants, for both before (AOR = 0.06; 95% CI 0.02, 0.16; *p* < 0.001) and after PSM (AOR = 0.06; 95% CI 0.01, 0.23; *p* < 0.001).

**Table 2:** Factors associated with dysmenorrhea according to BCC exposure status before and after propensity score matching using multivariable logistic regression.

Predictable Variables	AOR (95% CI) before propensity score matched		AOR (95% CI) after propensity score matched	
	Non-exposed (Control) (N = 238)	BCC-exposed (N = 234)	Non-exposed (Control) (N = 98)	BCC-exposed (N = 98)
***Physical activity***				
Sedentary (ref.)	–	–	–	–
Active and Athlete	0.25 (0.08, 0.78)*	0.23 (0.07, 0.68)**	0.15 (0.03, 0.89)*	0.06 (0.03, 0.40)**
***BMI (kg/m*** ^***2***^ **)**				
Normal weight (18.5–22.9) (ref.)	–	–	–	–
Underweight (< 18.5)	0.70 (0.19, 2.5)	1.8 (0.53, 6.2)	1.05 (0.19, 5.8)	0.75 (0.12, 4.8)
Overweight/obese (> 22.9)	2.06 (0.60, 7.06)	2.6 (0.60, 11.3)	5.3 (0.75, 37.6)	1.4 (0.28, 7.3)
***Dietary diversity score (DDS)***				
< 5 (Low) (ref.)	–	–	–	–
≥ 5 (High)	0.31 (0.10, 0.94)*	0.06 (0.02, 0.16)***	0.08 (0.01, 0.60)*	0.06 (0.01, 0.23)***
***Food craving (high fat and sweet food)***				
No (ref.)	–	–	–	–
Yes	1.4 (0.50, 4.0)	3.0 (1.1, 8.09)*	2.3 (0.45, 12.1)	2.1 (0.5, 9.2)
***Skipping breakfast***				
No (ref.)	–	–	–	–
Yes	0.80 (0.25, 2.6)	1.7 (0.66, 4.5)	0.88 (0.18, 4.2)	1.04 (0.27, 4.0)
***Sleep duration (hours)***				
≥ 7 hours per night (ref.)	–	–	–	–
< 7 hours per night	0.80 (0.28, 2.3)	3.5 (1.3, 9.1)*	0.38 (0.07, 1.9)	2.6 (0.63, 10.9)
***Caffeine consumption***				
Infrequent (< 3 times per week) (ref.)	–	–	–	–
Frequent (≥ 3 times per week)	1.1 (0.40, 3.3)	2.0 (0.75, 5.2)	0.60 (0.13, 2.8)	2.8 (0.65, 12.06)
***Family history of menstrual disorders***				
No (ref.)	–	–	–	–
Yes	2.4 (0.60, 9.8)	1.04 (0.30, 3.5)	1.9 (0.26, 14.01)	0.97 (0.22, 4.3)
***Age at menarche (years)***				
Mean (SD)	0.53 (0.36, 0.78)**	0.90 (0.66, 1.4)	0.60 (0.34, 1.04)	1.06 (0.71, 1.6)
***Marital status***				
Never married (ref.)	–	–	–	–
Ever married	0.50 (0.08, 3.08)	0.42 (0.01, 16.8)	1.4 (0.06, 31.9)	2.5 (0.01, 17.01)
***Father’s educational status***				
Secondary/higher (> 5 y schooling) (ref.)	–	–	–	–
Below secondary (0–5 y schooling)	7.5 (1.1, 49.8)*	1.6 (0.18, 14.5)	17.4 (0.48, 62.7)	1.0 (0.05, 16.8)
***Mother’s educational status***				
Secondary/higher (> 5 y schooling) (ref.)	–	–	–	–
Below secondary (0–5 y schooling)	0.18 (0.04, 1.4)	1.3 (0.27, 6.6)	0.18 (0.03, 1.3)	2.7 (0.30, 23.5)
***Mother’s occupational status***				
Formal occupation (ref.)	–	–	–	–
Informal occupation	0.68 (0.20, 2.7)	1.2 (0.36, 3.8)	1.15 (0.24, 5.5)	0.91 (0.18, 4.7)

AOR = adjusted odd ratio; BMI = body mass index; SD = standard deviation * indicated the level of significance, i.e., * p < 0.05, ** p < 0.01 and *** p < 0.001.

Certain lifestyle factors, such as food craving for high fat and sweet foods (AOR = 3.0; 95% CI 1.1, 8.09; *p* < 0.05) and inadequate sleep (< 7 hours per night, AOR = 3.5; 95% CI 1.3, 9.1; *p* < 0.05), were also associated with higher odds of dysmenorrhea in the unmatched sample among BCC-exposed participants. However, these associations were attenuated and no longer statistically significant in the matched analysis, suggesting that confounding factors may have influenced the initial observations.

### Association between BCC module exposure and dysmenorrhea

Table 3 exhibits the association between exposure to the BCC module, selected lifestyle factors, and dysmenorrhea before and after matching. Before matching, the odds ratio from the multivariable logistic regression model showed that participants who reported attending the BCC module had 87% lower odds of experiencing dysmenorrhea (AOR: 0.13, 95% CI: 0.06, 0.26; *p* < 0.001) compared with non-exposed participants. This association was adjusted for all covariates included in model 2, in addition to BCC module attendance. After PSM, the conditional (fixed-effect) logistic regression indicated a similar pattern, with BCC module exposure remaining significantly associated with a lower likelihood of dysmenorrhea (AOR: 0.13, 95% CI: 0.02, 0.79; *p* < 0.05), after controlling for the same set of covariates.

**Table 3:** Adjusted associations of BCC module exposure, key lifestyle factors, and dysmenorrhea before and after propensity score matching.

Variables	Before propensity score matching N = 472 (non-exposed = 238 and BCC-exposed = 234)		After propensity score matching N = 196 (Non-exposed = 98 and BCC-exposed = 98)
	Bivariate logistic model	Multivariable logistic model	Conditional logistic model (fixed-effect)
	COR (95% CI)	AOR (95% CI)	AOR (95% CI)
***Attended BCC module***			
No (ref.)	–	–	–
Yes	0.07 (0.04, 0.11)***	0.13 (0.06, 0.26)***	0.13 (0.02, 0.79)*
***Physical activity***			
Sedentary (ref.)	–	–	–
Active and Athlete	0.08 (0.05, 0.12)***	0.23 (0.11, 0.47)***	0.13 (0.02, 0.86)*
***Dietary diversity score (DDS)***			
< 5 (Low) (ref.)	–	–	–
≥ 5 (High)	0.10 (0.07, 0.16)***	0.12 (0.06, 0.23)***	0.06 (0.01, 0.32)**
***Food craving (high fat and sweet food)***			
No (ref.)	–	–	–
Yes	9.7 (6.2, 15.2)***	2.1 (1.08, 4.1)*	2.6 (2.07, 3.1)***

Crude odds ratios (COR) and adjusted odds ratios (AOR) with 95% confidence intervals (CI) were estimated using bivariate and multivariable logistic regression before propensity score matching. After matching, in the conditional (fixed-effect) logistic regression model, all co-variates matched in model 2 through PSM (physical activity, BMI, DDS, food cravings, skipping breakfast, sleep duration, caffeine consumption, family history of menstrual disorders, age at menarche, marital status, father’s education, mother’s education, and mother’s occupation) were adjusted together with BCC module exposure. Bootstrap standard errors (SE) for post-matching estimates were obtained using 500 replications with a fixed random seed (12345) to account for uncertainty in the estimated propensity scores. Statistical significance is identified as * p < 0.05, ** p < 0.01 and *** p < 0.001. The detailed results for all covariates are presented in S2 Table in S1 Data.

Furthermore, participants who were physically active or engaged in athletic activities showed a lower likelihood of experiencing dysmenorrhea in both pre- and post-matching analyses. Similarly, having a high dietary diversity score (DDS ≥ 5) was associated with lower odds of dysmenorrhea across all regression models. Conversely, food cravings for high fat and sweet foods were associated with a higher likelihood of dysmenorrhea before and after PSM. The detailed results for all covariates are presented in S2 Table in S1 Data. To further assess the robustness of the associations, we conducted sensitivity analyses using the full four-grade dysmenorrhea severity scale, as summarized in S3 Table in S1 Data.

### Sensitivity analysis

#### Sensitivity to outcome dichotomization.

The ordered logistic regression analysis (see details in S3 Table in S1 Data) showed results consistent with the primary dichotomized model. Attending the BCC module, being physically active and higher dietary diversity were significantly associated with lower odds of more severe dysmenorrhea; while shorter sleep duration, frequent caffeine consumption, and family history of menstrual disorders were associated with higher odds of more severe dysmenorrhea. However, most socio-demographic factors were not statistically significant. These findings indicate that the associations were robust when the full four-grade severity scale was used, suggesting that the results were not driven by outcome dichotomization.

#### Robustness to matching specifications.

Sensitivity analyses using alternative matching algorithms yielded consistent ATT estimates across all specifications (see details in S4 Table in S1 Data). Using the primary 1:1 nearest-neighbor matching with a caliper of 0.01, the adjusted difference in dysmenorrhea prevalence between the BCC-exposed and matched non-exposed groups was −0.23 (ATT = −0.23; 95% CI −0.36, −0.11; *p* < 0.001), indicating a 23 percentage-point lower prevalence in the BCC-exposed group compared with matched controls. ATT estimates remained stable when the number of matched neighbors increased, with −0.25 (95% CI −0.41, −0.08; *p* < 0.01) for 1:2 matching and −0.27 (95% CI −0.43, −0.12; *p* < 0.001) for 1:3 matching. Radius matching with wider calipers produced ATT estimates from −0.25 to −0.31, and kernel matching yielded −0.27 (95% CI −0.40, −0.13; *p* < 0.001). The consistency in direction and magnitude of these estimates across matching specifications indicates that observed differences are robust to the choice of matching algorithm.

#### Robustness to estimation approaches.

Table 4 represents additional sensitivity analyses using alternative estimation approaches to evaluate the robustness of the observed differences in dysmenorrhea prevalence between BCC-exposed and non-exposed participants. In the matched sample, the adjusted difference in dysmenorrhea prevalence estimated using IPWRA (doubly robust) was −0.23 (95% CI: −0.33 to −0.13; p < 0.001; Table 4), indicating a 23–percentage-point lower frequency of dysmenorrhea among BCC-exposed participants compared with matched non-exposed after adjusting for observed covariates. Estimates derived from RA (ATT = −0.23; 95% CI −0.33, −0.13; *p* < 0.001) and IPW (ATT = −0.23; 95% CI −0.34, −0.12; *p* < 0.001) were consistent with the doubly robust estimate, demonstrating stability across estimation methods.

**Table 4:** Adjusted differences in dysmenorrhea prevalence between BCC-exposed and non-exposed participants (ATT estimates).

Estimation approaches	ATT (95% CI) (N = 196)
Regression adjustment (RA)	−0.23 (−0.33, −0.13)***
Inverse probability weighting (IPW)	−0.23 (−0.34, −0.12)***
IPWRA (doubly robust)	−0.23 (−0.33, −0.13)***

ATT = Adjusted differences in dysmenorrhea prevalence between BCC-exposed and non-exposed participants after adjusted for observed covariates. Groups were made comparable using matching-based procedures, and estimates were then calculated using RA = Regression adjustment; IPW = Inverse probability weighting; or IPWRA = inverse probability weighting with regression adjustment (doubly robust estimator) methods. Robust standard errors (SE) are reported for each estimate (. Significance is indicated by * p < 0.05, ** p < 0.01, and *** p < 0.001.

Additional analyses incorporating propensity score matching with one or two neighbors as well as nearest-neighbor matching with and without bias adjustment produced ATT estimates similar to the primary doubly robust approach (see details in S5 Table in S1 Data), supporting the robustness of observed differences to alternative estimation methods.

#### Estimation of LBF.

In the unmatched sample, Bayesian logistic regression indicated that BCC module exposure was associated with lower odds of dysmenorrhea (OR = 0.13, 95% CrI: 0.06–0.24), with a LBF of 12.3, representing very strong statistical evidence supporting the presence of an association. In the matched sample, the association remained in the same direction (OR = 0.15, 95% CrI: 0.06–0.29), although the LBF decreased to 1.00, reflecting weaker statistical evidence. This reduction in evidential strength may be attributable to the smaller matched sample and improved covariate balance after matching. Physical activity and higher dietary diversity also showed consistent inverse associations with dysmenorrhea in both the unmatched and matched samples. In contrast, associations for several socio-demographic and lifestyle variables were attenuated in the matched sample (see details in S6 Table in S1 Data).

#### Impact of potential contamination on the observed association.

The observed AOR for the association between BCC module exposure and dysmenorrhea was 0.13 in the primary multivariable model. Under hypothetical contamination levels ranging from 10% to 50% among non-exposed participants, the corrected ORs progressively decreased from 0.10 to 0.02 (see details in S7 Table in S1 Data). Notably, even under extreme assumptions of 40–50% contamination, the corrected ORs remained substantially below 1.0. These findings suggest that potential indirect exposure among non-exposed participants would be more likely to reduce the magnitude of the observed association rather than fully explain it. Across all assumed contamination scenarios, the direction of association between BCC module exposure and dysmenorrhea remained consistently inverse.

## Discussion

In Bangladesh, this study is the first large-scale investigation describing the distribution of dysmenorrhea pain severity among female university students according to exposure to a BCC-based module. The findings revealed an association between BCC-exposed and a more favorable distribution of dysmenorrhea severity, with a lower proportion reporting mild, moderate, and severe pain and a higher proportion reporting no pain compared with non-exposed participants. These differences in pain distribution between the two groups were statistically significant prior to PSM, indicating an association between BCC module exposure and dysmenorrhea outcome after the six month follow-up. Although causal inference cannot be established due to the observational design and absence of pre-exposure outcome measurement, the observed patterns are consistent with the existing review literature suggesting that behavioral and lifestyle-oriented programs may be associated with improvements in dysmenorrhea-related symptoms when described as alternative or non-pharmacological treatments [2,3,13,25].

In line with these observed differences in pain distribution, further analysis explored lifestyle related factors that may contribute to dysmenorrhea outcomes among participants. The results of this study revealed that being physically active and having higher dietary diversity were associated with a lower prevalence of reported dysmenorrhea. Participants who reported being physically active and engaging in athletics and sports exhibited lower odds of reporting dysmenorrhea symptoms compared with sedentary participants, a pattern that is consistent with several prior studies [3,28,61]. Engaging in regular physical activity such as yoga, aerobic exercises, and strength training, promotes blood circulation to the pelvic area, alleviates cramping and enhances mood as well as stimulates the release of β-endorphins which act as non-specific analgesics [24,28]. Relaxation-based techniques such as aromatherapy massage, breathing, and meditation have also been associated with stress management and reductions in reported dysmenorrhea symptoms in some earlier studies [28,61]. However, compared with more intensive exercises, relaxation-oriented approaches have been noted to demonstrate favorable symptoms-related outcomes and have lower dropout rates [28]. Higher dietary diversity was also associated with lower odds of reporting dysmenorrhea in this study. Although a case-control study from Ethiopia stated no significant association between dietary diversity and dysmenorrhea [62], the patterns observed in the present study are consistent with findings from studies conducted in Saudi Arabia, Spain, and Jordan, which have reported associations between higher dietary diversity and more favorable dysmenorrhea-related symptom profiles as well as overall health indicators [26,63,64]. Additionally, a systematic review of observational studies further supports these findings, indicating that a healthier dietary pattern, characterized by higher intakes of fruits and vegetables as sources of vitamins and minerals, along with fish, milk, and dairy products, is associated with a lower prevalence of reported dysmenorrhea [27].

Furthermore, several studies have highlighted the association between unhealthy dietary habits, such as frequent consumption of sugary foods, sweets, and ultra-processed or junk food, and higher levels of reported dysmenorrhea severity [26,64,65]. These observations are consistent with our findings, which indicate that participants reporting stronger cravings for high-fat and sugary foods was associated with higher odds of experiencing dysmenorrhea symptoms. Taken together, these patterns highlight the relevance of dietary behaviors and food preferences in relation to menstrual pain experiences.

Additionally, our study observed that shorter sleep duration (< 7 hours per night) had higher odds of experiencing dysmenorrhea symptoms. This finding aligns with the evidence from systemic review and meta-analysis by Mitsuhashi et al. [37], which reported associations between sleep characteristics and menstrual pain. Similarly, Woosley et al. found that participants with mild dysmenorrhea tended to have better sleep quality compared with those experiencing moderate or severe symptoms [66]. These observations collectively suggest a potential association between dysmenorrhea and sleep disturbances, highlighting the possible relevance of sleep patterns in relation to menstrual pain experiences. Nevertheless, the available evidence does not clarify the directionality of this relationship.

Beyond these lifestyle correlates, this study also examined whether participation in the BCC module was associated with differences in dysmenorrhea outcomes. The findings suggest a consistent association between participation in the BCC module and lower prevalence and severity of dysmenorrhea pain among female university students. In the initial bivariate analyses, participants exposed to the BCC module had substantially lower odds of reporting dysmenorrhea compared with those not exposed. This association persisted even after adjusting for socio-demographic, lifestyle, or dietary covariates in multivariable logistic regression, suggesting that the measured factors did not fully explain the observed relationship. Although the magnitude of the association was slightly attenuated after adjustment, the direction and statistical significance remained unchanged, supporting the consistency of the findings across analytic approaches.

To further address concerns related to confounding and selection bias inherent in non-randomized studies, PSM was applied to improve comparability between the BCC-exposed and non-exposed groups. Conditional logistic regression conducted on the matched sample yielded results similar to those from multivariable regression models, indicating that the association between BCC module exposure and dysmenorrhea was stable across different analytic strategies. However, residual confounding due to unmeasured factors cannot be excluded. Currently, these findings cannot be compared with previous studies, as there is limited research specifically examining the BCC-based programs in relation to dysmenorrhea. Earlier studies have primarily focused on single component strategies, particularly exercise-based approaches [28,61,67,68] or dietary modifications [27,69] which have demonstrated beneficial associations with dysmenorrhea symptoms. Although these approaches target modifiable lifestyle factors, their single-focus nature may be insufficient to sustain long-term behavior change or address broader determinants of menstrual health [70]. Approaches for addressing dysmenorrhea symptoms may benefit from comprehensive and multicomponent programs. As highlighted by Laverack (2017), such programs can integrate behavior change strategies, promote supportive environments through policy frameworks, and empower individuals to make informed lifestyle choices [70]. Evidence from a systematic review also suggests that interventions targeting both diet and physical activity may be associated with more favorable outcomes when they incorporated well-defined behavior change techniques such as goal-setting and self-monitoring [71]. In line with this evidence, the present study observed an association between attendance at the BCC module and a lower prevalence of dysmenorrhea, alongside a greater likelihood of being physically active and having higher dietary diversity. These associations were consistent across all regression models, indicating stability in the observed patterns. However, these findings should be interpreted with caution, as the observational nature of the study does not allow causal inferences. The results do not establish that the BCC module directly caused behavioral change or improvements in menstrual outcomes, but they align with patterns expected from theory-driven, structured, multicomponent BCC programs that may promote healthier behaviors.

Building on the primary analysis, we also conducted sensitivity analyses using alternative matching algorithms and estimation approaches to further support the robustness of the observed association between BCC exposure and dysmenorrhea. ATT estimates remained consistent across nearest-neighbor matching with varying numbers of neighbors, radius matching with wider calipers, kernel matching, as well as IPWRA, RA, and IPW estimators. The magnitude and direction of the association remained stable across these specifications, indicating that the observed association was not dependent on a particular matching algorithm or modeling strategy. Methodological literature on propensity score analysis indicates that consistency across alternative matching strategies provides stronger support for internal validity rather than reliance on a single specification, particularly in non-randomized studies [72,73]. Similarly, Mondal et al. also reported reliable ATT estimates for BCC-exposed participants [74]. While the reduced matched sample may have limited the ability to detect very small associations, consistent estimates across multiple sensitivity analyses indicate that the restrictions resulting from the strict caliper did not materially alter the overall pattern of association. This reflects a deliberate methodological choice to prioritize internal validity and covariate balance over sample size, ensuring more precise and comparable matches between BCC-exposed and non-exposed participants.

To complement these analyses, Bayesian estimation was conducted, confirming that the inverse association between BCC module participation, physical activity, higher dietary diversity, and dysmenorrhea was directionally consistent with the results from multivariable regression and propensity score based approaches. Although the evidence appeared stronger in the unmatched sample, this evidence was attenuated after PSM, reflecting a more balanced comparison between the groups. Overall, the Bayesian findings offer complementary support for the observed pattern, rather than serving as standalone proof of effect magnitude.

Finally, it is important to recognize that the composition of the matched sample can vary depending on which covariates are included in the propensity score model, even when the matching algorithm itself remains fixed [72,42]. In observational analyses, differences in covariate selection may affect the magnitude and precision of estimated group differences, particularly when balance is incomplete [73]. Methodological guidance emphasizes the importance of pre-specifying covariate selection strategies and balance criteria to reduce analytic flexibility and the risk of specification searching [42,75]. While a structured and predefined approach was applied in the present study, findings from propensity score methods remain inherently dependent on modeling assumptions and the variables included in the score calculation, highlighting that the results may vary with different covariate selections or model choices [72,73]. Accordingly, the findings should be interpreted within this analytical context.

### Strengths and limitations

This study has several methodological strengths that enhance the credibility of the observed findings. It examined associations within a structured, theory-informed BCC framework implemented in a real-world university setting. The data quality in this study is considered high, with trained female supervisors and data collectors engaged to foster to create a comfortable environment for discussing menstruation-related issues and to minimize bias in understanding technical terms. Additionally, the analysis incorporated key covariates identified in the systematic reviews by Mitsuhashi et al. and Greaves et al. as relevant to behavior adherence [37,71], improving adjustment for measured confounders. Propensity score matching resulted in improved balanced across observed covariates, and multiple analytic approaches produced directionally similar results, indicating stability of the observed associations across statistical methods. Participants were followed up for six months, a standard duration for assessing intermediate-term behavioral patterns, which allowed sufficient time to integrate new habits compared with shorter follow-ups [71,76]. The BCC module was guided by the theory of change framework emphasizing awareness, motivation, skill building and self-efficacy. While this framework provides conceptual justification for examining behavioral associations, the present design does not allow determination of whether these components directly produced the report outcomes.

Despite these strengths, several important limitations must be emphasized. First, baseline prevalence of dysmenorrhea was not measured prior to BCC exposure, preventing assessment of temporal sequencing and causal inference. Although, propensity score matching improves comparability on measured covariates, it cannot replace baseline outcome measurements. Therefore, the observed differences may reflect pre-existing variation between groups, and self-selection bias and regression to the mean remain plausible alternative explanations. Thus, the study should be interpreted as a cross-sectional comparison between exposed and non-exposed groups rather than as an evaluation of intervention effectiveness. Second, the non-randomized design introduces potential selection bias. Despite improved balance on observed characteristics through matching, residual confounding from unmeasured or unknown factors cannot be excluded. Third, the strict caliper applied in propensity score matching resulted in a smaller matched sample (98 pairs), which may have reduced statistical power. However, consistent results across sensitivity analyses suggesting that the reduced sample did not materially compromise the robustness of the findings. Fourth, contamination between groups may have affected internal validity, as exposed and non-exposed participants were recruited from the same university and dormitory settings, allowing potential peer interactions and indirect exposure to BCC-related information. No formal monitoring or empirical verification of non-exposure was conducted. Therefore, the extent of contamination cannot be quantified. Any indirect exposure could have attenuated differences between groups. Sensitivity analyses explored hypothetical contamination scenarios, but these remain assumption based. Fifth, dysmenorrhea was assessed using self-reported Andersch-Milsom multidimensional scoring system, which has not been formally validated in Bangladeshi population. Formal inter-rater reliability was not calculated and outcome assessors were not blinded to the group allocation, which may introduce potential measurement bias. Nevertheless, structured questionnaires and predefined scoring criteria were used to reduce subjective variation. Even though the attendance at BCC sessions was documented, these records do not capture the intensity of participant engagement or to the extent to which recommended behaviors were implemented. Furthermore, detailed quantitative dietary intake data were unavailable. Although, dietary data were collected using a five-day 24-hour recall based on FAO guidelines, recall bias may have affected accuracy and the representation of true dietary habits. Finally, the study was conducted among female university students in a specific setting, which may limit the generalizability of the findings to other populations or contexts. Overall, the findings indicate an association between BCC module exposure and reported dysmenorrhea patterns. However, given the absence of baseline outcome data, potential self-selection, non-random design, and possible contamination, the result should be interpreted as associative rather causal.

## Conclusion

This study identified a consistent association between exposure to a BCC module and lower reported prevalence of dysmenorrhea among female university in Bangladesh. Participants who attended BCC sessions also more likely to report engaging in physical activity and maintaining higher dietary diversity, indicating that multi-component, theory-driven BCC approach was associated with lifestyle behaviors relevant to the management of dysmenorrhea. These findings highlight the potential importance of structured behavior change programs implemented within university settings to promote healthier lifestyle practices that may support menstrual health. However, due to the absence of baseline dysmenorrhea measurements and non-randomized matched cross-sectional design, causal inferences cannot be drown and the findings should be interpreted as association rather than the intervention effectiveness. Further longitudinal or randomized studies are needed to determine whether the structured BCC programs can directly influence menstrual health outcomes and to better understand the mechanisms linking lifestyle behaviors and dysmenorrhea.

In addition, strengthening supportive on-campus environment such as access to nutritious food options, opportunities for physical activity, and adequate recreational space may further help to promote healthy behaviors among university students and support the improvement in menstrual health.

## Supporting information

S1 Data S1 Appendix. Structure and content of the BCC module. S2 Appendix. Logic model of the BCC module guided by Transtheoretical model (stage of change). S1 File. Informed consent form (ICF). S2 File. Questionnaire in English version. S3 File. Database. S1A Table. Covariate balance before and after propensity score matching under alternative pre-specified model specification (means, %bias, percentage bias reduction, t-test and variance ratios). S1B Table. Overall balance statistics (Rubin’s B and Rubin’s R) under pre-specified propensity score specifications. S2 Table. Adjusted associations of BCC module exposure and key lifestyle factors with dysmenorrhea before and after propensity score matching. S3 Table. Sensitivity analysis: Ordered logistic regression assessing associations of BCC exposure and covariates with four-grade dysmenorrhea severity (unmatched sample, N = 472). S4 Table. Sensitivity analysis of dysmenorrhea prevalence differences under alternative propensity score matching algorithms and specifications. S5 Table. Sensitivity analysis: Adjusted differences in dysmenorrhea prevalence across multiple analytic approaches (ATT and ATE estimates). S6 Table. Sensitivity analysis: Bayesian logistic regression analysis for dysmenorrhea comparing models with and without BCC module exposure. S7 Table. Sensitivity analysis: Corrected adjusted odds ratios (ORs) for the BCC exposure under assumed levels of contamination among non-exposed participants. S1 Fig. Original pamphlet for behavioral change communication (BCC) module. S2 Fig. Distribution of BCC-exposed and non-exposed (control) observations according to whether they are “on support” or “off support” after matching. S1 Text. Calculation of the sample size and proportional distribution among the universities. S2 Text. Explanation of the outcome variable. S3 Text. Detailed information of each covariate. S4 Text. Estimation of BCC associated differences (ATT and ATE estimates) using propensity score matching. S5 Text. Detail calculation of the Log Bayes Factor (LBF). S6 Text. Sensitivity analysis for potential contamination.
(ZIP)
