# Supplementary material for: Association between attendance at a behavioral change communication module and dysmenorrhea prevalence among female university students: A propensity score matched comparative study
Source: PLoS One. 2026 May 12;21(5):e0349064. doi: 10.1371/journal.pone.0349064 (PMC13166925; doi:10.1371/journal.pone.0349064)
Supplement: S1 Data — S2 Appendix. Logic model of the BCC module guided by Transtheoretical model (stage of change). S1 File. Informed consent form (ICF). S2 File. Questionnaire in English version. S3 File. Database. S1A Table. Covariate balance before and after propensity score matching under alternative pre-specified model specification (means, %bias, percentage bias reduction, t-test and variance ratios). S1B Table. Overall balance statistics (Rubin’s B and Rubin’s R) under pre-specified propensity score specifications. S2 Table. Adjusted associations of BCC module exposure and key lifestyle factors with dysmenorrhea before and after propensity score matching. S3 Table. Sensitivity analysis: Ordered logistic regression assessing associations of BCC exposure and covariates with four-grade dysmenorrhea severity (unmatched sample, N = 472). S4 Table. Sensitivity analysis of dysmenorrhea prevalence differences under alternative propensity score matching algorithms and specifications. S5 Table. Sensitivity analysis: Adjusted differences in dysmenorrhea prevalence across multiple analytic approaches (ATT and ATE estimates). S6 Table. Sensitivity analysis: Bayesian logistic regression analysis for dysmenorrhea comparing models with and without BCC module exposure. S7 Table. Sensitivity analysis: Corrected adjusted odds ratios (ORs) for the BCC exposure under assumed levels of contamination among non-exposed participants. S1 Fig. Original pamphlet for behavioral change communication (BCC) module. S2 Fig. Distribution of BCC-exposed and non-exposed (control) observations according to whether they are “on support” or “off support” after matching. S1 Text. Calculation of the sample size and proportional distribution among the universities. S2 Text. Explanation of the outcome variable. S3 Text. Detailed information of each covariate. S4 Text. Estimation of BCC associated differences (ATT and ATE estimates) using propensity score matching. S5 Text. Detail calculation of the Log Bayes Factor (LBF). [file pone.0349064.s001.zip › supporting materials/S1 Appendix.docx]

**S1 Appendix: Structure and content of the BCC module**

Each participant group attended three structured sessionsa corresponding to the three core domains of the Behavior Change Communication (BCC) module. All sessions followed a standardized lesson plan and were delivered by trained female educators using a facilitator’s manual to ensure consistency across groups and study sites. Each session combined short lectures, interactive discussions, demonstrations, and goal-setting activities to enhance understanding, engagement, and behavioral intention.

**Session one: Menstrual disorders and associated risk factors**

This session focused on developing participants’ understanding of menstrual health and the common characteristics of menstrual disorders. Basic menstrual physiology and the normal menstrual cycle were explained in simple terms. Common menstrual disorders, with particular emphasis on dysmenorrhea, were discussed in relation to both biological and lifestyle-related factors.

Participants were introduced to a range of modifiable and non-modifiable risk factors associated with menstrual disorders. These included poor dietary diversity, frequent consumption of high-fat and high-sugar foods (food cravings), physical inactivity, overweight and obesity, psychological stress, insufficient sleep, excessive caffeine intake, early age at menarche, and family history of menstrual problems. The links between these factors and the severity or frequency of menstrual symptoms were described to enhance awareness of personal risk.

Common myths, misconceptions, and cultural stigmas surrounding menstruation were addressed and clarified to encourage open discussion and healthy attitudes toward menstrual health. Participants were encouraged to reflect on their own experiences and to identify personal and environmental factors that might contribute to menstrual discomfort. The session concluded with an interactive discussion and a brief knowledge-check activity to reinforce key concepts and clarify misunderstandings.

**Session two: Lifestyle modification with emphasis on dietary habits**

The second session focused on the importance of healthy dietary practices in supporting menstrual well-being. Participants received guidance on the role of a balanced and diverse diet, regular meals, and adequate hydration in maintaining overall health and reducing menstrual discomfort. An overview of major food groups was provided, along with explanations of how these foods contribute to bodily function and wellbeing.

Special attention was given to key micronutrients relevant to women’s health, including vitamin A, vitamin B-complex, vitamin C, iron, and calcium. Their roles in supporting energy levels, blood health, bone health, and hormonal balance were explained using practical and culturally appropriate food examples that were locally available and affordable.

Participants were advised to limit the intake of highly processed foods, foods high in sugar and unhealthy fats, salty snacks, and caffeinated beverages. They were encouraged to assess their current eating patterns, identify unhealthy habits, and set realistic, achievable goals for improvement. Small group activities were used to help participants develop simple and practical meal ideas that could be incorporated into their daily routines.

**Session Three: Physical activity and supportive lifestyle practices**

The third session highlighted the role of regular physical activity and other supportive lifestyle practices in managing menstrual discomfort and enhancing overall well-being. Participants were informed about the benefits of physical activity for pain reduction, stress management, improved circulation, and emotional well-being.

Simple stretching exercises, breathing techniques, and selected yoga postures suitable for practice in dormitory or home environments were demonstrated and practiced during the session. Participants were encouraged to perform these movements regularly and to select activities that were realistic and enjoyable within their daily schedules.

In addition to physical activity, other supportive lifestyle factors were discussed, including the importance of maintaining a healthy body weight, improving sleep quality, managing stress effectively, and reducing unhealthy food cravings through balanced nutrition and healthy coping strategies.

The session concluded with a short reflection and goal-setting activity, in which participants identified specific actions they intended to take to improve their physical activity levels, manage stress, and support their overall menstrual health.

**Standardization and Quality Assurance**

All sessions were delivered using uniform educational materials, including presentation slides, printed handouts, and a facilitator’s guide. The educators followed a standardized structure that included an introduction, content delivery, interactive discussion, practical demonstration (yoga), and reinforcement of key messages. Attendance was recorded for each session, and participant engagement was encouraged through questions, discussions, and brief activities. Ongoing communication between the educators and the research team ensured fidelity to the intervention protocol across all sites.

**Adherence and Reinforcement Mechanisms**

Participants received reminders before each session and were provided with brief take-home messages summarizing the main points. At the end of each session, participants set personal lifestyle goals related to dietary habits, physical activity, hydration, and other supportive practices. During the follow-up phase, these goals were revisited and reinforced through periodic supportive contact with the educators to encourage continued engagement in positive behavior change.
