# Supplementary material for: Association between attendance at a behavioral change communication module and dysmenorrhea prevalence among female university students: A propensity score matched comparative study
Source: PLoS One. 2026 May 12;21(5):e0349064. doi: 10.1371/journal.pone.0349064 (PMC13166925; doi:10.1371/journal.pone.0349064)
Supplement: S1 Data — S2 Appendix. Logic model of the BCC module guided by Transtheoretical model (stage of change). S1 File. Informed consent form (ICF). S2 File. Questionnaire in English version. S3 File. Database. S1A Table. Covariate balance before and after propensity score matching under alternative pre-specified model specification (means, %bias, percentage bias reduction, t-test and variance ratios). S1B Table. Overall balance statistics (Rubin’s B and Rubin’s R) under pre-specified propensity score specifications. S2 Table. Adjusted associations of BCC module exposure and key lifestyle factors with dysmenorrhea before and after propensity score matching. S3 Table. Sensitivity analysis: Ordered logistic regression assessing associations of BCC exposure and covariates with four-grade dysmenorrhea severity (unmatched sample, N = 472). S4 Table. Sensitivity analysis of dysmenorrhea prevalence differences under alternative propensity score matching algorithms and specifications. S5 Table. Sensitivity analysis: Adjusted differences in dysmenorrhea prevalence across multiple analytic approaches (ATT and ATE estimates). S6 Table. Sensitivity analysis: Bayesian logistic regression analysis for dysmenorrhea comparing models with and without BCC module exposure. S7 Table. Sensitivity analysis: Corrected adjusted odds ratios (ORs) for the BCC exposure under assumed levels of contamination among non-exposed participants. S1 Fig. Original pamphlet for behavioral change communication (BCC) module. S2 Fig. Distribution of BCC-exposed and non-exposed (control) observations according to whether they are “on support” or “off support” after matching. S1 Text. Calculation of the sample size and proportional distribution among the universities. S2 Text. Explanation of the outcome variable. S3 Text. Detailed information of each covariate. S4 Text. Estimation of BCC associated differences (ATT and ATE estimates) using propensity score matching. S5 Text. Detail calculation of the Log Bayes Factor (LBF). [file pone.0349064.s001.zip › supporting materials/S1 Fig.docx]

**Behavioral changes for the management of menstrual disorders**

**Prevalence of Menstrual Disorders**
Among female university students in Bangladesh, common menstrual disorders include dysmenorrhea (68.3%), premenstrual syndrome (PMS) (33.8%) and irregular menstrual cycle (IMC) (24.3%).

**Dysmenorrhea Overview**
Dysmenorrhea refers to painful menstrual cramps originating in the uterus, often experienced during menstruation.

***Symptoms***

- Cramping pain in the lower abdomen, radiating to the back or thighs
- Pain starting 1-2 days before menstruation and lasting for several days
- Associated symptoms: nausea, vomiting, diarrhea, fatigue, and headaches

***Physical Consequences***

- Chronic pain may increase sensitivity to pain over time
- Fatigue due to managing pain and related symptoms
- Untreated underlying conditions (e.g., endometriosis) may worsen secondary dysmenorrhea

***Emotional and Psychological Impact***

- Stress and anxiety: Anticipation of pain can heighten stress levels
- Depression: Chronic pain may lead to feelings of hopelessness
- Low self-esteem: Persistent symptoms can affect self-image

***Impact on Education and Work***

- Reduced productivity due to pain and discomfort
- Increased absenteeism from school or work
- Career limitations in physically demanding or high-pressure fields

***Reproductive Health Complications***

- Infertility: Conditions like endometriosis can affect fertility
- Compromised quality of life: Persistent pain lowers overall well-being

**Treatment and Management**
Lifestyle changes can significantly improve symptoms, including:

- Dietary adjustments: Eat a balanced diet rich in vitamin A from plant sources; avoid high-fat and sugary foods
- Regular physical activity: Engage in yoga or other exercises for at least 30 minutes, 4 days a week
- Healthy habits: Avoid skipping breakfast and maintain ≥7 hours of sleep nightly. Limit caffeine consumption

Adopting these behaviors can enhance menstrual health and overall quality of life.

**The right way to change behavior**

**Step 1: Set a Goal and Start Date**

Choose a clear goal and commit to starting immediately

**Step 2: Prepare for Change**

Reflect on the need for a healthier lifestyle and develop a specific action plan within the next six months. Keep in mind that the first 15 days can be challenging, so stay committed.

**Step 3: Implement Behavioral Modifications**
Within the next 30 days, follow your action plan by adopting these practices:

- Eat a balanced diet, including at least 5 food groups daily
- Engage in regular physical exercise, such as practicing yoga for 30 minutes/day, 4 days a week
- Avoid high-fat and sugary foods, and limit caffeine intake
- Never skip breakfast
- Sleep at leaast 7 hours per night

**Step 4: Sustain Behavioral Practices (0–6 Months)**

Continue these new habits for less than six months, avoiding situations that may disrupt your progress such as

- Avoid triggers: Stay away from fast food locations or street vendors
- Practice mindful eating: Enjoy your meals but focus on variety, not just rice
- Choose quality over quantity: Prioritize nutritional value over taste or portion size
- Follow the "Three D Principles": Healthy diet, disciplined lifestyle, and necessary drugs

**Step 5: Maintain Long-Term Change (6+ Months)**

To maintain healthy behaviors beyond six months, use these strategies to stay motivated:

- Plan weekly meals to ensure consistent healthy choices
- Incorporate short walking or stretching breaks into your routine
- Set small, achievable daily goals to keep progressing
- Remind yourself regularly of the benefits
- Connect with others who share similar health goals for support
- Try new, healthy recipes
- Use a journal or apps to track your meals, exercise, and progress
- Keep healthy snacks like yogurt, nuts, and fruits readily available
- Practice stress reduction techniques, such as meditation or deep breathing
- Explore fun activities (i.e. cycling, dancing)
- Place motivational quotes or pictures in visible areas to inspire you daily
- Work with a friend for accountability and enjoyment in maintaining habits

**Step 6: Advocate for a Healthy Lifestyle**

After six months, become a role model and advocate for a healthy lifestyle:

- Regularly practice your new habits.
- Share the benefits of your transformation with others to inspire and motivate them.

The pamphlet actually used in written in Bangla language, but the content is the same in this English description

**S1 Fig. Original pamphlet for behavioral change communication (BCC) module**
