# Supplementary material for: Association between attendance at a behavioral change communication module and dysmenorrhea prevalence among female university students: A propensity score matched comparative study
Source: PLoS One. 2026 May 12;21(5):e0349064. doi: 10.1371/journal.pone.0349064 (PMC13166925; doi:10.1371/journal.pone.0349064)
Supplement: S1 Data — S2 Appendix. Logic model of the BCC module guided by Transtheoretical model (stage of change). S1 File. Informed consent form (ICF). S2 File. Questionnaire in English version. S3 File. Database. S1A Table. Covariate balance before and after propensity score matching under alternative pre-specified model specification (means, %bias, percentage bias reduction, t-test and variance ratios). S1B Table. Overall balance statistics (Rubin’s B and Rubin’s R) under pre-specified propensity score specifications. S2 Table. Adjusted associations of BCC module exposure and key lifestyle factors with dysmenorrhea before and after propensity score matching. S3 Table. Sensitivity analysis: Ordered logistic regression assessing associations of BCC exposure and covariates with four-grade dysmenorrhea severity (unmatched sample, N = 472). S4 Table. Sensitivity analysis of dysmenorrhea prevalence differences under alternative propensity score matching algorithms and specifications. S5 Table. Sensitivity analysis: Adjusted differences in dysmenorrhea prevalence across multiple analytic approaches (ATT and ATE estimates). S6 Table. Sensitivity analysis: Bayesian logistic regression analysis for dysmenorrhea comparing models with and without BCC module exposure. S7 Table. Sensitivity analysis: Corrected adjusted odds ratios (ORs) for the BCC exposure under assumed levels of contamination among non-exposed participants. S1 Fig. Original pamphlet for behavioral change communication (BCC) module. S2 Fig. Distribution of BCC-exposed and non-exposed (control) observations according to whether they are “on support” or “off support” after matching. S1 Text. Calculation of the sample size and proportional distribution among the universities. S2 Text. Explanation of the outcome variable. S3 Text. Detailed information of each covariate. S4 Text. Estimation of BCC associated differences (ATT and ATE estimates) using propensity score matching. S5 Text. Detail calculation of the Log Bayes Factor (LBF). [file pone.0349064.s001.zip › supporting materials/S1 File.docx]

**S1 File. Informed consent form (ICF)**

**Research project Title:** **Effectiveness of Behavioral Change Communication Intervention in Dysmenorrhea Management among Female University Students: A Propensity Score Matching Analysis**

Objective of the Study: to assess the effectiveness of a behavioral change communication intervention in managing dysmenorrhea among female university students in Bangladesh.

*(The interviewer will introduce herself and take consent of the interviewee before commencing the session)*

**Introductory statement:**

My name is……………. I am currently working for Mr. Liton Chandra Sen, Associate Professor at the Faculty of Nutrition and Food Science, Patuakhali Science and Technology University. I have been assigned as an interviewer for the aforementioned PhD project, conducted under the Department of Food Engineering and Tea Technology at Shahjalal University of Science and Technology. Therefore, I kindly request your permission to conduct the interview, which typically takes about 15–20 minutes. We assure you that your identity will not be disclosed in any reports derived from this interview. Your confidentiality will be strictly maintained. You may skip any question or stop the interview at any time. With your consent, I would like to take written notes during the interview.

**What will you need to do if you agree to participate?**

As a selected respondent, your valuable input on a few key issues is requested. If any question makes you feel uncomfortable, you are free to skip it or not respond.

**Risks, Benefits, Confidentiality and compensation for participating**

There are no risks to participating. Your input will support Mr. Sen’s PhD research and help improve national reproductive health programs for young adults. All information will remain strictly confidential and used only for research by authorized personnel. Your participation in this study is completely voluntary and offers no financial compensation. There is no pressure or obligation to take part.

**Right to Refuse or Withdraw**
This study has been approved by the Ethical Approval Committee at Shahjalal University of Science and Technology. Participation is voluntary, and you can skip questions or withdraw at any time.

**Who do I contact if I have a question or problem?**

If you wish to know more about your rights as a participant in this study you may write Mr. Liton Chadra Sen, Associate Professor, Department of Community Health and Hygiene, Patuakhali Science and Technology University, Dumki, patuakhali-8602, email- liton.sen@pstu.ac.bd, mobile no. +8801717504808. If you have further questions regarding the nature of this study you may also contact Professor Dr. G.M. Rabiul Islam, Department of Food Engineering and Tea Technology, Shahajalal University of Science and Technology, Sylhet, email- [rabiat14@yahoo.com](mailto:rabiat14@yahoo.com), phone: 880 821 713 850 extn.242 (office); Cell: +88 01787323944.

**Do you have any questions about the interview?**May we begin now? If so, please read and sign the consent form, and keep a copy for your records.

**Consent to publication:**

This is to state that I give my full permission for the publication, reproduction, broadcast and other use of identifiable details, which can include case history and/or details within the text (schedule A, B, C, D and E) to be published in any Journal and Article. I confirm that I have seen and been given the opportunity to read the methodology to be published by any journal.

I understand that the published article may be available in both print and on the internet, and will be available to a broader audience through marketing channels and other third parties. Therefore, anyone can read material published in the Journal. I understand that readers may include not only public health professionals and scholarly researchers but also journalists and general members of the public. I also understand that the information will be published without my personal details and every attempt will be made to ensure anonymity.

I declare, in consequence of granting this permission, that I have no claim on ground of breach of confidence or any other ground in any legal system against the authors and its agents, publishers, successors and assigns in respect of such use of the collected information.

Participant’s Name: ………………………………… Signature ……………….. Date: …………

Name of person obtaining consent: ………………… Signature: ……………… Date: ………..

*(Must be study investigator or individual who has been designated to obtain consent)*

*To be counter-signed and dated electronically for telephone interviews or in the presence of the participant for face to face interviews.*
