# Supplementary material for: Association between attendance at a behavioral change communication module and dysmenorrhea prevalence among female university students: A propensity score matched comparative study
Source: PLoS One. 2026 May 12;21(5):e0349064. doi: 10.1371/journal.pone.0349064 (PMC13166925; doi:10.1371/journal.pone.0349064)
Supplement: S1 Data — S2 Appendix. Logic model of the BCC module guided by Transtheoretical model (stage of change). S1 File. Informed consent form (ICF). S2 File. Questionnaire in English version. S3 File. Database. S1A Table. Covariate balance before and after propensity score matching under alternative pre-specified model specification (means, %bias, percentage bias reduction, t-test and variance ratios). S1B Table. Overall balance statistics (Rubin’s B and Rubin’s R) under pre-specified propensity score specifications. S2 Table. Adjusted associations of BCC module exposure and key lifestyle factors with dysmenorrhea before and after propensity score matching. S3 Table. Sensitivity analysis: Ordered logistic regression assessing associations of BCC exposure and covariates with four-grade dysmenorrhea severity (unmatched sample, N = 472). S4 Table. Sensitivity analysis of dysmenorrhea prevalence differences under alternative propensity score matching algorithms and specifications. S5 Table. Sensitivity analysis: Adjusted differences in dysmenorrhea prevalence across multiple analytic approaches (ATT and ATE estimates). S6 Table. Sensitivity analysis: Bayesian logistic regression analysis for dysmenorrhea comparing models with and without BCC module exposure. S7 Table. Sensitivity analysis: Corrected adjusted odds ratios (ORs) for the BCC exposure under assumed levels of contamination among non-exposed participants. S1 Fig. Original pamphlet for behavioral change communication (BCC) module. S2 Fig. Distribution of BCC-exposed and non-exposed (control) observations according to whether they are “on support” or “off support” after matching. S1 Text. Calculation of the sample size and proportional distribution among the universities. S2 Text. Explanation of the outcome variable. S3 Text. Detailed information of each covariate. S4 Text. Estimation of BCC associated differences (ATT and ATE estimates) using propensity score matching. S5 Text. Detail calculation of the Log Bayes Factor (LBF). [file pone.0349064.s001.zip › supporting materials/S1 Text.docx]

**S1 Text. Calculation of the sample size and proportional distribution among the universities**

A proportional 1:1 sample size was calculated for an observational study comparing two independent groups defined by participation in a Behavior Change Communication (BCC) module. Participants were categorized based on a dichotomous response: “Yes” for those who attended the module (BCC-exposed group) and “No” for those who did not attend (non-exposed group). The comparison group comprised non-participants and was used to assess differences in menstrual health outcomes between exposure groups.

The anticipated prevalence of menstrual disorders in the non-exposed group was 42% 42% [[1](#_ENREF_1)]. The anticipated prevalence of menstrual disorders in the non-exposed group was 42% [1]. The sample size was calculated to detect a 30% relative difference in prevalence between the BCC-exposed and non-exposed groups. Based on a two-sided 95% confidence level and 80% statistical power, the required total sample size was estimated to be 452 participants, with 226 in each group [[2](#_ENREF_2), [3](#_ENREF_3)]. This calculation was based on a 95% confidence interval (CI) and 80% power.

***The calculation of the sample size:***

n$=\frac{\left( Z_{\propto/2}+Z_{\beta} \right)^{2} \times\left[ P_{1}\left( 1-P_{1} \right) +P_{2}\left( 1-P_{2} \right) \right]}{\left( P_{1}- P_{2} \right)^{2}}$

n$=$Required sample size per group

$P_{1}=$ 0.42 (42% anticipated prevalence of the outcome in the non-exposed group)

$P_{2}=$ 0.294 (expected 30% relative difference from 42% in the BCC-exposed group)

$$Z_{\propto/2}=1.96 (Z value for 95\% confidence interval)$$

$$Z_{\beta}= 0.84 (Z value corresponding to 80\% power)$$

*Calculation of the variance for both groups:*

- Control (non-exposed) group = $P_{1}\left( 1-P_{1} \right)$ = 0.42 $\times$ (1 - 0.42) = 0.25
- BCC-exposed group = $P_{2}\left( 1-P_{2} \right)$ = 0.294 $\times$(1 - 0.294) = 0.21

*Sum of the variances of both groups:*

$P_{1}\left( 1-P_{1} \right)$ + $P_{2}\left( 1-P_{2} \right)$ $=$0.25 + 0.21 = 0.46

*Calculation of the difference in proportions:*

$P_{1}- P_{2}=$0.42−0.294=0.126

Now, finally these calculation into the sample size formula:

n$=\frac{\left( 1.96 +0.84 \right)^{2} \times0.46}{\left( 0.126 \right)^{2}}$

n$=\frac{\left( 2.8 \right)^{2} \times0.46}{0.016}$

n$=\frac{7.84 \times0.46}{0.016}=$ $\frac{3.61}{0.016}$ = 225.6 $\approx$ 226

Thus, the required sample size was **226 participants in each group**, resulting in a total estimated sample size of:

N (Total) = 226 $\times2=452$

***Proportional distribution of total sample among the universities:***

The proportional distribution of the total sample of 452 (236 per group) among Patuakhli Science and Technology University (PSTU), Barisal University (BU) and Khulna University (KU) based on the total populations as follows:

Proportional sample $= \frac{Univerity population}{Total populaton} \times$ Total sample size

*The total population across the three universities:*

Total population = 1311(PSTU) + 2783(BU) + 1204(KU) = 5298

**1*.* BCC-exposed group (n=236)**

PSTU: $\frac{1131}{5298}$ $\times$ 226 = 55.9 $\approx56$

BU: $\frac{2783}{5298}$ $\times$ 226 = 118.7 $\approx119$

KU: $\frac{1204}{5298}$ $\times$ 226 = 51.3 $\approx51$

**2. Non-exposed group (n=236)**

PSTU: $\frac{1131}{5298}$ $\times$ 226 = 55.9 $\approx56$

BU: $\frac{2783}{5298}$ $\times$ 226 = 118.7 $\approx119$

KU: $\frac{1204}{5298}$ $\times$ 226 = 51.3 $\approx51$

**Final Proportional Distribution**

- **PSTU:** 56 BCC-exposed, 56 non-exposed
- **BU:** 119 BCC-exposed, 119 non-exposed
- **KU:** 51 BCC-exposed, 51 non-exposed

Total = 452 participants.

**Reference**

1. Sen LC, Jahan I, Salekin N, Shourove JH, Rahman M, Uddin MJ, et al. Food craving, vitamin A, and menstrual disorders: A comprehensive study on university female students*.* PloS one. 2024; **19**(9):e0310995. 10.1371/journal.pone.0310995 PMID: PMID

2. Cohen J. Statistical power analysis for the behavioral sciences. 2nd Edition ed. 2013, New York: routledge.

3. Liu XS. Statistical power analysis for the social and behavioral sciences: Basic and advanced techniques. 1st Edition ed. 2013, New York: Routledge.
