# Supplementary material for: Association between attendance at a behavioral change communication module and dysmenorrhea prevalence among female university students: A propensity score matched comparative study
Source: PLoS One. 2026 May 12;21(5):e0349064. doi: 10.1371/journal.pone.0349064 (PMC13166925; doi:10.1371/journal.pone.0349064)
Supplement: S1 Data — S2 Appendix. Logic model of the BCC module guided by Transtheoretical model (stage of change). S1 File. Informed consent form (ICF). S2 File. Questionnaire in English version. S3 File. Database. S1A Table. Covariate balance before and after propensity score matching under alternative pre-specified model specification (means, %bias, percentage bias reduction, t-test and variance ratios). S1B Table. Overall balance statistics (Rubin’s B and Rubin’s R) under pre-specified propensity score specifications. S2 Table. Adjusted associations of BCC module exposure and key lifestyle factors with dysmenorrhea before and after propensity score matching. S3 Table. Sensitivity analysis: Ordered logistic regression assessing associations of BCC exposure and covariates with four-grade dysmenorrhea severity (unmatched sample, N = 472). S4 Table. Sensitivity analysis of dysmenorrhea prevalence differences under alternative propensity score matching algorithms and specifications. S5 Table. Sensitivity analysis: Adjusted differences in dysmenorrhea prevalence across multiple analytic approaches (ATT and ATE estimates). S6 Table. Sensitivity analysis: Bayesian logistic regression analysis for dysmenorrhea comparing models with and without BCC module exposure. S7 Table. Sensitivity analysis: Corrected adjusted odds ratios (ORs) for the BCC exposure under assumed levels of contamination among non-exposed participants. S1 Fig. Original pamphlet for behavioral change communication (BCC) module. S2 Fig. Distribution of BCC-exposed and non-exposed (control) observations according to whether they are “on support” or “off support” after matching. S1 Text. Calculation of the sample size and proportional distribution among the universities. S2 Text. Explanation of the outcome variable. S3 Text. Detailed information of each covariate. S4 Text. Estimation of BCC associated differences (ATT and ATE estimates) using propensity score matching. S5 Text. Detail calculation of the Log Bayes Factor (LBF). [file pone.0349064.s001.zip › supporting materials/S1A Table.docx]

**S1A Table. Covariate balance before and after propensity score matching under alternative pre-specified model specification (means, %bias, percentage bias reduction, t-test and variance ratios)**

| **Model ID** | **Covariates** | **Unmatched/matched** | **Mean** | | **% Bias** | **Percentage bias Reduction** | **T-test** | | **V(T)/V(C**) |
| --- | --- | --- | --- | --- | --- | --- | --- | --- | --- |
|  |  |  | **Control (Non-exposed)** | **BCC exposed** |  |  | **t** | ***p* > t** |  |
| Model 1 | Physical activity | U | 0.87 | 0.49 | -88.2 |  | -9.6 | 0.000 | . |
|  |  | M | 0.81 | 0.85 | 8.3 | 90.6 | 0.7 | 0.479 | . |
|  | BMI (Kg/m^2^) | U | 2.3 | 2 | -50.6 |  | -5.5 | 0.000 | 0.47* |
|  |  | M | 2 | 2 | 0.0 | 100.0 | 0.0 | 1.000 | 1.04 |
|  | Dietary diversity score (DDS) (≥ 5) | U | 0.64 | 0.41 | -47.8 |  | -5.2 | 0.000 | . |
|  |  | M | 0.55 | 0.51 | -9.2 | 80.8 | -0.7 | 0.505 | . |
|  | Age at menarche | U | 12.1 | 13.4 | 75.4 |  | 8.2 | 0.000 | 1.25 |
|  |  | M | 12.6 | 12.7 | 8.3 | 89.0 | 0.7 | 0.511 | 1.07 |
| Model 2 | Physical activity | U | 0.87 | 0.49 | -88.2 |  | -9.6 | 0.000 | . |
|  |  | M | 0.79 | 0.77 | -4.8 | 94.6 | -0.3 | 0.734 | . |
|  | BMI (Kg/m^2^) | U | 2.3 | 2 | -50.6 |  | -5.5 | 0.000 | 0.47* |
|  |  | M | 2.03 | 2.06 | 4.7 | 90.7 | 0.3 | 0.759 | 0.79 |
|  | Dietary diversity score (DDS) (≥ 5) | U | 0.64 | 0.41 | -47.8 |  | -5.2 | 0.000 | . |
|  |  | M | 0.53 | 0.55 | 4.2 | 91.2 | 0.3 | 0.776 | . |
|  | Food craving (high fat and sweet food) | U | 0.68 | 0.3 | -81.3 |  | -8.8 | 0.000 | . |
|  |  | M | 0.54 | 0.53 | -2.2 | 97.3 | -0.1 | 0.887 | . |
|  | Skipping breakfast | U | 0.7 | 0.28 | -92.3 |  | -10.0 | 0.000 | . |
|  |  | M | 0.51 | 0.53 | 4.5 | 95.1 | 0.3 | 0.776 | . |
|  | Sleep duration (hours) | U | 0.68 | 0.34 | -73.9 |  | -8.0 | 0.000 | . |
|  |  | M | 0.55 | 0.57 | 4.3 | 94.1 | 0.3 | 0.775 | . |
|  | Caffeine consumption (frequency per week) | U | 0.73 | 0.33 | -86.7 |  | -9.4 | 0.000 | . |
|  |  | M | 0.57 | 0.6 | 6.7 | 92.3 | 0.4 | 0.665 | . |
|  | Family history of menstrual disorders | U | 0.33 | 0.16 | -40.3 |  | -4.4 | 0.000 | . |
|  |  | M | 0.3 | 0.28 | -4.8 | 88.0 | -0.3 | 0.753 | . |
|  | Age at menarche (years) | U | 12.1 | 13.4 | 75.4 |  | 8.2 | 0.000 | 1.25 |
|  |  | M | 12.6 | 12.6 | 0.6 | 99.2 | 0.0 | 0.966 | 1.15 |
|  | Marital status | U | 0.09 | 0.03 | -24.0 |  | -2.6 | 0.009 | . |
|  |  | M | 0.05 | 0.04 | -4.2 | 82.5 | -0.3 | 0.735 | . |
|  | Father’s educational status | U | 0.21 | 0.07 | -40.2 |  | -4.4 | 0.000 | . |
|  |  | M | 0.11 | 0.12 | 3.0 | 92.6 | 0.2 | 0.825 | . |
|  | Mother’s educational status | U | 0.26 | 0.17 | -23.0 |  | -2.5 | 0.013 | . |
|  |  | M | 0.2 | 0.21 | 2.5 | 89.1 | 0.2 | 0.861 | . |
|  | Mother’s occupational status | U | 0.72 | 0.81 | 21.2 |  | 2.3 | 0.022 | . |
|  |  | M | 0.71 | 0.76 | 9.7 | 54.3 | 0.6 | 0.520 | . |
| Model 3 | Physical activity | U | 0.87 | 0.49 | -88.2 |  | -9.6 | 0.000 | . |
|  |  | M | 0.76 | 0.78 | 5.3 | 94.0 | 0.4 | 0.721 | . |
|  | BMI (Kg/m^2^) | U | 2.3 | 2 | -50.6 |  | -5.5 | 0.000 | 0.47* |
|  |  | M | 2.1 | 2.07 | -17.5 | 65.4 | -1.1 | 0.264 | 0.91 |
|  | Dietary diversity score (DDS) (≥ 5) | U | 0.64 | 0.41 | -47.8 |  | -5.2 | 0.000 | . |
|  |  | M | 0.56 | 0.56 | 0.0 | 100.0 | 0.0 | 1.000 | . |
|  | Food craving (high fat and sweet food) | U | 0.68 | 0.3 | -81.3 |  | -8.8 | 0.000 | . |
|  |  | M | 0.6 | 0.59 | -2.4 | 97.0 | -0.2 | 0.879 | . |
|  | Skipping breakfast | U | 0.7 | 0.28 | -92.3 |  | -10.0 | 0.000 | . |
|  |  | M | 0.55 | 0.55 | 0.0 | 100.0 | 0.0 | 1.000 | . |
|  | Sleep duration (hours) | U | 0.68 | 0.34 | -73.9 |  | -8.0 | 0.000 | . |
|  |  | M | 0.51 | 0.56 | 9.7 | 86.9 | 0.6 | 0.548 | . |
|  | Caffeine consumption (frequency per week) | U | 0.73 | 0.33 | -86.7 |  | -9.4 | 0.000 | . |
|  |  | M | 0.61 | 0.63 | 2.5 | 97.1 | 0.2 | 0.877 | . |
|  | Bedtime | U | 0.89 | 0.41 | -117.6 |  | -12.8 | 0.000 | . |
|  |  | M | 0.76 | 0.77 | 2.8 | 97.7 | 0.2 | 0.859 | . |
|  | Family history of menstrual disorders | U | 0.33 | 0.16 | -40.3 |  | -4.4 | 0.000 | . |
|  |  | M | 0.22 | 0.24 | 5.4 | 86.6 | 0.4 | 0.721 | . |
|  | Age at menarche (years) | U | 12.1 | 13.4 | 75.4 |  | 8.2 | 0.000 | 1.25 |
|  |  | M | 12.4 | 12.3 | -4.9 | 93.5 | -0.4 | 0.729 | 1.12 |
|  | Marital status | U | 0.09 | 0.03 | -24.0 |  | -2.6 | 0.009 | . |
|  |  | M | 0.05 | 0.05 | 0.0 | 100.0 | 0.0 | 1.000 |  |
|  | Father’s educational status | U | 0.21 | 0.07 | -40.2 |  | -4.4 | 0.000 | . |
|  |  | M | 0.1 | 0.13 | 6.6 | 83.5 | 0.5 | 0.637 | . |
|  | Mother’s educational status | U | 0.26 | 0.17 | -23.0 |  | -2.5 | 0.013 | . |
|  |  | M | 0.17 | 0.19 | 5.6 | 75.8 | 0.4 | 0.698 | . |
|  | Mother’s occupational status | U | 0.72 | 0.81 | 21.2 |  | 2.3 | 0.022 | . |
|  |  | M | 0.76 | 0.77 | 2.7 | 87.3 | 0.2 | 0.859 | . |
| Model 4 | Physical activity | U | 0.87 | 0.49 | -88.2 |  | -9.6 | 0.000 | . |
|  |  | M | 0.76 | 0.78 | 4.9 | 94.4 | 0.3 | 0.733 | . |
|  | BMI (Kg/m^2^) | U | 2.3 | 2 | -50.6 |  | -5.5 | 0.000 | 0.47* |
|  |  | M | 2.03 | 2 | -4.9 | 90.4 | -0.3 | 0.755 | 0.86 |
|  | Dietary diversity score (DDS) (≥ 5) | U | 0.64 | 0.41 | -47.8 |  | -5.2 | 0.000 | . |
|  |  | M | 0.53 | 0.52 | -2.2 | 95.5 | -0.1 | 0.885 | . |
|  | Food craving (high fat and sweet food) | U | 0.68 | 0.3 | -81.3 |  | -8.8 | 0.000 | . |
|  |  | M | 0.58 | 0.56 | -4.5 | 94.4 | -0.3 | 0.771 | . |
|  | Skipping breakfast | U | 0.7 | 0.28 | -92.3 |  | -10.0 | 0.000 | . |
|  |  | M | 0.56 | 0.53 | -6.9 | 92.5 | -0.4 | 0.664 | . |
|  | Caffeine consumption (frequency per week) | U | 0.73 | 0.33 | -86.7 |  | -9.4 | 0.000 | . |
|  |  | M | 0.57 | 0.61 | 9.2 | 89.4 | 0.6 | 0.558 | . |
|  | Age at menarche (years) | U | 12.1 | 13.4 | 75.4 |  | 8.2 | 0.000 | 1.25 |
|  |  | M | 12.5 | 12.6 | 3.9 | 94.8 | 0.3 | 0.790 | 1.03 |
|  | Father’s educational status | U | 0.21 | 0.07 | -40.2 |  | -4.4 | 0.000 | . |
|  |  | M | 0.11 | 0.14 | 9.2 | 77.0 | 0.7 | 0.507 | . |
| Model 5 | Physical activity | U | 0.87 | 0.49 | -88.2 |  | -9.6 | 0.000 | . |
|  |  | M | 0.76 | 0.8 | 9.4 | 89.3 | 0.7 | 0.497 | . |
|  | BMI (Kg/m^2^) | U | 2.3 | 2 | -50.6 |  | -5.5 | 0.000 | 0.47* |
|  |  | M | 2.05 | 2.07 | 3.1 | 93.9 | 0.2 | 0.833 | 0.91 |
|  | Dietary diversity score (DDS) (≥ 5) | U | 0.64 | 0.41 | -47.8 |  | -5.2 | 0.000 | . |
|  |  | M | 0.51 | 0.51 | 0.0 | 100.0 | 0.0 | 1.000 | . |
|  | Skipping breakfast | U | 0.7 | 0.28 | -92.3 |  | -10.0 | 0.000 | . |
|  |  | M | 0.52 | 0.48 | -6.7 | 92.8 | -0.4 | 0.672 | . |
|  | Caffeine consumption (frequency per week) | U | 0.73 | 0.33 | -86.7 |  | -9.4 | 0.000 | . |
|  |  | M | 0.55 | 0.53 | -4.4 | 94.9 | -0.3 | 0.777 | . |
|  | Age at menarche (years) | U | 12.1 | 13.4 | 75.4 |  | 8.2 | 0.000 | 1.25 |
|  |  | M | 12.7 | 12.5 | -8.8 | 88.4 | -0.6 | 0.530 | 1.12 |
|  | Father’s educational status | U | 0.21 | 0.07 | -40.2 |  | -4.4 | 0.000 | . |
|  |  | M | 0.14 | 0.13 | -3.0 | 92.7 | -0.2 | 0.837 | . |
|  | Mother’s educational status | U | 0.26 | 0.17 | -23.0 |  | -2.5 | 0.013 | . |
|  |  | M | 0.22 | 0.18 | -9.9 | 56.9 | -0.7 | 0.481 | . |
| Model 6 | Physical activity | U | 0.87 | 0.49 | -88.2 |  | -9.6 | 0.000 | . |
|  |  | M | 0.77 | 0.77 | 0.0 | 100.0 | 0.0 | 1.000 | . |
|  | BMI (Kg/m^2^) | U | 2.3 | 2 | -50.6 |  | -5.5 | 0.000 | 0.47* |
|  |  | M | 2.07 | 2.03 | -6.4 | 87.4 | -0.4 | 0.674 | 0.77 |
|  | Dietary diversity score (DDS) (≥ 5) | U | 0.64 | 0.41 | -47.8 |  | -5.2 | 0.000 | . |
|  |  | M | 0.53 | 0.57 | 8.5 | 82.3 | 0.6 | 0.566 | . |
|  | Food craving (high fat and sweet food) | U | 0.68 | 0.3 | -81.3 |  | -8.8 | 0.000 | . |
|  |  | M | 0.47 | 0.51 | 6.7 | 91.8 | 0.4 | 0.669 | . |
|  | Skipping breakfast | U | 0.7 | 0.28 | -92.3 |  | -10.0 | 0.000 | . |
|  |  | M | 0.55 | 0.52 | -6.8 | 92.6 | -0.4 | 0.668 | . |
|  | Sleep duration (hours) | U | 0.68 | 0.34 | -73.9 |  | -8.0 | 0.000 | . |
|  |  | M | 0.56 | 0.57 | 2.2 | 97.0 | 0.1 | 0.886 | . |
|  | Age at menarche (years) | U | 12.1 | 13.4 | 75.4 |  | 8.2 | 0.000 | 1.25 |
|  |  | M | 12.8 | 12.7 | -7.0 | 90.7 | -0.5 | 0.606 | 0.98 |
|  | Father’s educational status | U | 0.21 | 0.07 | -40.2 |  | -4.4 | 0.000 | . |
|  |  | M | 0.09 | 0.12 | 9.0 | 77.5 | 0.7 | 0.491 | . |
|  | Mother’s educational status | U | 0.09 | 0.03 | -24.0 |  | -2.6 | 0.009 | . |
|  |  | M | 0.05 | 0.06 | 4.3 | 82.3 | 0.3 | 0.758 | . |
|  | Mother’s occupational status | U | 0.26 | 0.17 | -23.0 |  | -2.5 | 0.013 | . |
|  |  | M | 0.15 | 0.2 | 10.1 | 56.1 | 0.8 | 0.453 | . |
| Model 7 | Physical activity | U | 0.87 | 0.49 | -88.2 |  | -9.6 | 0.000 | . |
|  |  | M | 0.78 | 0.77 | -2.4 | 97.3 | -0.2 | 0.866 | . |
|  | BMI (Kg/m^2^) | U | 2.3 | 2 | -50.6 |  | -5.5 | 0.000 | 0.47* |
|  |  | M | 2.01 | 2.03 | 3.1 | 93.8 | 0.2 | 0.838 | 0.92 |
|  | Dietary diversity score (DDS) (≥ 5) | U | 0.64 | 0.41 | -47.8 |  | -5.2 | 0.000 | . |
|  |  | M | 0.53 | 0.55 | 4.2 | 91.2 | 0.3 | 0.776 | . |
|  | Food craving (high fat and sweet food) | U | 0.68 | 0.3 | -81.3 |  | -8.8 | 0.000 | . |
|  |  | M | 0.48 | 0.55 | 15.4 | 81.1 | 1.0 | 0.320 | . |
|  | Skipping breakfast | U | 0.7 | 0.28 | -92.3 |  | -10.0 | 0.000 | . |
|  |  | M | 0.54 | 0.52 | -4.5 | 95.1 | -0.3 | 0.776 | . |
|  | Caffeine consumption (frequency per week) | U | 0.73 | 0.33 | -86.7 |  | -9.4 | 0.000 | . |
|  |  | M | 0.58 | 0.6 | 4.4 | 94.9 | 0.3 | 0.773 | . |
|  | Age at menarche (years) | U | 12.1 | 13.4 | 75.4 |  | 8.2 | 0.000 | 1.25 |
|  |  | M | 12.5 | 12.6 | -1.3 | 98.3 | -0.1 | 0.931 | 1.06 |
|  | Father’s educational status | U | 0.21 | 0.07 | -40.2 |  | -4.4 | 0.000 | . |
|  |  | M | 0.15 | 0.14 | -3.00 | 92.60 | -0.20 | 0.842 | . |
|  | Mother’s educational status | U | 0.26 | 0.17 | -23.00 |  | -2.50 | 0.013 | . |
|  |  | M | 0.23 | 0.22 | -2.50 | 89.10 | -0.17 | 0.866 | . |

BMI = body mass index; * If variance ratio outside [0.77; 1.29] for U and [0.67; 1.49] for M
