# Supplementary material for: Association between attendance at a behavioral change communication module and dysmenorrhea prevalence among female university students: A propensity score matched comparative study
Source: PLoS One. 2026 May 12;21(5):e0349064. doi: 10.1371/journal.pone.0349064 (PMC13166925; doi:10.1371/journal.pone.0349064)
Supplement: S1 Data — S2 Appendix. Logic model of the BCC module guided by Transtheoretical model (stage of change). S1 File. Informed consent form (ICF). S2 File. Questionnaire in English version. S3 File. Database. S1A Table. Covariate balance before and after propensity score matching under alternative pre-specified model specification (means, %bias, percentage bias reduction, t-test and variance ratios). S1B Table. Overall balance statistics (Rubin’s B and Rubin’s R) under pre-specified propensity score specifications. S2 Table. Adjusted associations of BCC module exposure and key lifestyle factors with dysmenorrhea before and after propensity score matching. S3 Table. Sensitivity analysis: Ordered logistic regression assessing associations of BCC exposure and covariates with four-grade dysmenorrhea severity (unmatched sample, N = 472). S4 Table. Sensitivity analysis of dysmenorrhea prevalence differences under alternative propensity score matching algorithms and specifications. S5 Table. Sensitivity analysis: Adjusted differences in dysmenorrhea prevalence across multiple analytic approaches (ATT and ATE estimates). S6 Table. Sensitivity analysis: Bayesian logistic regression analysis for dysmenorrhea comparing models with and without BCC module exposure. S7 Table. Sensitivity analysis: Corrected adjusted odds ratios (ORs) for the BCC exposure under assumed levels of contamination among non-exposed participants. S1 Fig. Original pamphlet for behavioral change communication (BCC) module. S2 Fig. Distribution of BCC-exposed and non-exposed (control) observations according to whether they are “on support” or “off support” after matching. S1 Text. Calculation of the sample size and proportional distribution among the universities. S2 Text. Explanation of the outcome variable. S3 Text. Detailed information of each covariate. S4 Text. Estimation of BCC associated differences (ATT and ATE estimates) using propensity score matching. S5 Text. Detail calculation of the Log Bayes Factor (LBF). [file pone.0349064.s001.zip › supporting materials/S1B Table.docx]

**S1B Table. Overall balance statistics (Rubin’s B and Rubin’s R) under pre-specified propensity score specifications**

| **Model ID** | **Adjusted covariates for each model** | **Ps R²** | **LR Chi²** | **Log Likelihood** | **Mean Bias (Matched)** | **B (Matched)** | **R (Matched)** | **Number of balanced covariates within ± 10% bias** | **Matched sample**  **(BCC-exposed/non-exposed)** |
| --- | --- | --- | --- | --- | --- | --- | --- | --- | --- |
| Model 1 | physical activity, BMI, DDS, and age at menarche | 0.19 | 121.2 | -266.6 | 6.5 | 15.8 | 0.85 | 4 out of 4 | 112/112 |
| Model 2 | physical activity, BMI, DDS, food craving (high-fat and sweet foods), skipping breakfast, sleep duration (hours), caffeine consumption (frequency per week), family history of menstrual disorders, age at menarche (years), marital status, father’s educational status, mother’s educational status, and mother’s occupational status | 0.33 | 214.3 | -220.0 | 4.3 | 17.3 | 0.80 | 13 out of 13 | 98/98 |
| Model 3 | physical activity, BMI, DDS, food craving (high-fat and sweet foods), skipping breakfast, sleep duration (hours), caffeine consumption (frequency per week), bedtime, family history of menstrual disorders, age at menarche (years), marital status, father’s educational status, mother’s educational status, and mother’s occupational status | 0.36 | 236.1 | -209.1 | 4.7 | 24.7 | 0.75 | 13 out of 14 | 88/88 |
| Model 4 | physical activity, BMI, DDS, food craving (high-fat and sweet foods), skipping breakfast, caffeine consumption (frequency per week), age at menarche (years), and father’s educational status | 0.30 | 197.0 | -228.7 | 5.7 | 18.7 | 0.68 | 8/ out of 8 | 95/95 |
| Model 5 | physical activity, BMI, DDS, skipping breakfast, caffeine consumption (frequency per week), age at menarche (years), father’s educational status and mother’s educational status | 0.29 | 192.4 | -231.0 | 5.7 | 19.3 | 1.05 | 8 out of 8 | 99/99 |
| Model 6 | physical activity, BMI, DDS, food craving (high-fat and sweet foods), skipping breakfast, sleep duration (hours), age at menarche (years), father’s educational status, mother’s educational status, and mother’s occupational status | 0.30 | 199.5 | -227.4 | 6.1 | 19.8 | 0.84 | 9 out of 10 | 97/97 |
| Model 7 | physical activity, BMI, DDS, food craving (high-fat and sweet foods), skipping breakfast, caffeine consumption (frequency per week), age at menarche (years), father’s educational status, and mother’s educational status | 0.30 | 197.2 | -228.6 | 4.5 | 16.5 | 1.06 | 8 out of 9 | 98 /98 |

*Rubin’s* ***B****: Standardized difference in mean propensity scores between BCC-exposed and non-exposed groups and acceptable if < 25. Rubin’s* ***R****: Ratio of variances of propensity scores between the study groups and acceptable if between 0.5 and 2.0. * If B>25%, R outside [0.5; 2]. BMI = Body mass index and DDS= Dietary diversity score*
