# Supplementary material for: Association between attendance at a behavioral change communication module and dysmenorrhea prevalence among female university students: A propensity score matched comparative study
Source: PLoS One. 2026 May 12;21(5):e0349064. doi: 10.1371/journal.pone.0349064 (PMC13166925; doi:10.1371/journal.pone.0349064)
Supplement: S1 Data — S2 Appendix. Logic model of the BCC module guided by Transtheoretical model (stage of change). S1 File. Informed consent form (ICF). S2 File. Questionnaire in English version. S3 File. Database. S1A Table. Covariate balance before and after propensity score matching under alternative pre-specified model specification (means, %bias, percentage bias reduction, t-test and variance ratios). S1B Table. Overall balance statistics (Rubin’s B and Rubin’s R) under pre-specified propensity score specifications. S2 Table. Adjusted associations of BCC module exposure and key lifestyle factors with dysmenorrhea before and after propensity score matching. S3 Table. Sensitivity analysis: Ordered logistic regression assessing associations of BCC exposure and covariates with four-grade dysmenorrhea severity (unmatched sample, N = 472). S4 Table. Sensitivity analysis of dysmenorrhea prevalence differences under alternative propensity score matching algorithms and specifications. S5 Table. Sensitivity analysis: Adjusted differences in dysmenorrhea prevalence across multiple analytic approaches (ATT and ATE estimates). S6 Table. Sensitivity analysis: Bayesian logistic regression analysis for dysmenorrhea comparing models with and without BCC module exposure. S7 Table. Sensitivity analysis: Corrected adjusted odds ratios (ORs) for the BCC exposure under assumed levels of contamination among non-exposed participants. S1 Fig. Original pamphlet for behavioral change communication (BCC) module. S2 Fig. Distribution of BCC-exposed and non-exposed (control) observations according to whether they are “on support” or “off support” after matching. S1 Text. Calculation of the sample size and proportional distribution among the universities. S2 Text. Explanation of the outcome variable. S3 Text. Detailed information of each covariate. S4 Text. Estimation of BCC associated differences (ATT and ATE estimates) using propensity score matching. S5 Text. Detail calculation of the Log Bayes Factor (LBF). [file pone.0349064.s001.zip › supporting materials/S2 Appendix.docx]

**S2 Appendix. Logic model of the BCC module guided by Transtheoretical model (stage of change)**

| **Inputs (Resources)** | **Activities (TTM-Aligned Components)** | **Outputs** | **Short-Term Outcomes (0–3 months)** | **Medium-Term Outcomes (3–6 months)** | **Long-Term Impact (>6 months)** |
| --- | --- | --- | --- | --- | --- |
| **Human Resources**   - Two trained female educators - Expert guidance from a community health professor and a gynecologist - Three yoga trainers | **Pre-contemplation / Contemplation**   - Increase awareness of menstrual disorders, dysmenorrhea, and associated lifestyle determinants - Correct misconceptions - Enhance perceived benefits of healthy behaviors | 39 structured BCC sessions delivered across three core domains:   - menstrual disorders and risk factors - lifestyle/dietary modification - physical activity and supportive lifestyle practices | Increased awareness of menstrual disorders, dysmenorrhea and self-care behaviors | Sustained engagement in physical activity, yoga, hydration and stress-management strategies | Improved menstrual health and overall quality of life |
| **Standardized BCC Materials**   - Multimedia presentations - Pamphlets - Quizzes | **Preparation**   - Facilitate personal goal-setting for diet, physical activity, hydration, sleep quality, and stress reduction - Identify individual barriers and plan feasible behavior change strategies. | Educational materials distributed | Improved attitudes toward healthy dietary and physical activity practices | Adoption of balanced diet, improved dietary diversity, and regular physical activity | Continued adherence to healthy lifestyle behaviors |
| **Engagement & Logistical Supports**   - Dormitory meeting space for small groups - Light refreshments (vanilla cake + apple) - Small gift (sanitary napkin) - Certificate of participation | **Action**   - Conduct interactive sessions: dietary planning, physical activity/yoga demonstrations - Guided discussions to promote weight management, sleep hygiene, hydration, craving control, and stress-reduction practices | Increased peer-group engagement and interactive learning | Initiation of goal-directed behavior change | Maintenance of healthy lifestyle routines | A scalable TTM-based intervention model for university settings |
| **Implementation Supports**   - SMS reminder system - Supervision and fidelity monitoring by educators under a research supervisor | **Maintenance**   - Conduct reinforcement visits every two months for six month follow-up - Provide support for sustaining behavior change, problem-solving challenges, and encouraging adherence | Increased exposure to reinforcement messages and follow-up support | Enhanced self-efficacy and readiness to progress through stages of change | Reduced severity and frequency of dysmenorrhea | Contributes to broader women’s health promotion in Bangladesh |
