# Supplementary material for: Association between attendance at a behavioral change communication module and dysmenorrhea prevalence among female university students: A propensity score matched comparative study
Source: PLoS One. 2026 May 12;21(5):e0349064. doi: 10.1371/journal.pone.0349064 (PMC13166925; doi:10.1371/journal.pone.0349064)
Supplement: S1 Data — S2 Appendix. Logic model of the BCC module guided by Transtheoretical model (stage of change). S1 File. Informed consent form (ICF). S2 File. Questionnaire in English version. S3 File. Database. S1A Table. Covariate balance before and after propensity score matching under alternative pre-specified model specification (means, %bias, percentage bias reduction, t-test and variance ratios). S1B Table. Overall balance statistics (Rubin’s B and Rubin’s R) under pre-specified propensity score specifications. S2 Table. Adjusted associations of BCC module exposure and key lifestyle factors with dysmenorrhea before and after propensity score matching. S3 Table. Sensitivity analysis: Ordered logistic regression assessing associations of BCC exposure and covariates with four-grade dysmenorrhea severity (unmatched sample, N = 472). S4 Table. Sensitivity analysis of dysmenorrhea prevalence differences under alternative propensity score matching algorithms and specifications. S5 Table. Sensitivity analysis: Adjusted differences in dysmenorrhea prevalence across multiple analytic approaches (ATT and ATE estimates). S6 Table. Sensitivity analysis: Bayesian logistic regression analysis for dysmenorrhea comparing models with and without BCC module exposure. S7 Table. Sensitivity analysis: Corrected adjusted odds ratios (ORs) for the BCC exposure under assumed levels of contamination among non-exposed participants. S1 Fig. Original pamphlet for behavioral change communication (BCC) module. S2 Fig. Distribution of BCC-exposed and non-exposed (control) observations according to whether they are “on support” or “off support” after matching. S1 Text. Calculation of the sample size and proportional distribution among the universities. S2 Text. Explanation of the outcome variable. S3 Text. Detailed information of each covariate. S4 Text. Estimation of BCC associated differences (ATT and ATE estimates) using propensity score matching. S5 Text. Detail calculation of the Log Bayes Factor (LBF). [file pone.0349064.s001.zip › supporting materials/S2 File.docx]

**S2 File. Questionnaire in english version**

**Schedule A: personal detail of the participant**

| Name of the participant |  | | | |
| --- | --- | --- | --- | --- |
| Participant’s group based on BCC exposure |  | BCC-exposed group |  | Non-exposed (control) group |
| Name of the University |  | | | |
| Home District |  | | | |
| Mobile number (if any) |  | | | |

**Schedule B: Demographic and clinical information**

| **Residence** | **Age** | **Age at Menarche** | **Marital Status** | **Contraceptive use** |
| --- | --- | --- | --- | --- |
| 1 | 2 | 3 | 4 | 5 |
| Usually where do you live?   1. With family 2. At student dormitory | How old are you?  (Years) | At what age did your menstruation start for first time?  (Years) | What is your current marital status?   1. Never married 2. Ever married | If answer is ever married according to column 5 then-  What type of contraceptive method do you use?  (Write the method name) |
|  |  |  |  |  |

| **Parent’s educational status** | **Parent’s occupation** | **Body Mass Index**  **(BMI)** | | | | **Physical activity** |
| --- | --- | --- | --- | --- | --- | --- |
| 6 | 7 | 8 | | | | 9 |
| What is your parent’s educational status?  1. Below secondary  (0-5 y schooling)  2. Secondary/Higher  (>5 y schooling)  (Write down 2 digits, 1^st^ for father and 2^nd^ for mother education) | What do your parent currently do?  1. Formal occupation  2. Informal occupation  (Write down 2 digits, 1^st^ for father and 2^nd^ for mother education) | Calculate the BMI by measuring the height (cm) and weight (kg)  (Use first cell for height and second cell for weight) | | | | What type of Physical activity do you perform?  1.Sedentary (typical activities with little or no exercise)  2. Active (moderate-intensity activities such as brisk walking, light jogging, or moderate exercise e.g. yoga, weight lifting etc. for at least 150–300 minutes per week)  3. Athlete (vigorous-intensity activities running, fast cycling, jumping, climbing, swimming or competitive sports etc. for at least 75-150 minutes per week) |
|  |  |  | inch |  | kg |  |

**Schedule C: Assessing the severity of dysmenorrhea pain and consequences**

| Sl  No. | Questions and filters | Coding categories | Code number/answer |
| --- | --- | --- | --- |
| 1. | **Assessment of Dysmenorrhea**  (Whether the menstrual period is painful, affect daily activity and requires analgesics) | **Grade 0:** not painful and daily activity is unaffected  **Grade1:** painful but seldom inhibits normal activity; analgesics are seldom required; mild pain  **Grade 2:** Daily activity is affected; analgesics required and give sufficient relief so that absence from school is unusual; moderate pain  **Grade 3:** Activity clearly inhibited; poor effect of analgesics; vegetative symptoms (headache, fatigue, vomiting, and diarrhea); severe pain |  |
| 2. | **Menstrual disorder history**  Do you have any family history (mother and sister) of menstrual disorders? | 1. No 2. Yes |  |
| 3. | **Missed class**  Did you miss your class in last 2 months because of a menstrual disorder? | 0. No  1. Yes |  |

**Schedule D: Life style factors**

| Sl  No. | Questions and filters | Coding categories | Code number/answer |
| --- | --- | --- | --- |
| 1. | **Food craving**  Do you like to consume high fat and sugar foods? | 1. No 2. Yes |  |
| 2. | **Breakfast skipping**  Did you skip breakfast 1 or more times in the last week? | 1. No 2. Yes |  |
| 3. | **Sleep duration (hours)**  For how many hours do you sleep per night? | 1. <7 hours per night  2. ≥ 7 hours per night |  |
| 4. | **Bedtime**  At what time do you go to bed? | 1. 23:00 and before 2. After 23:00 |  |
| 5. | **Caffeine consumption**  Did you intake caffeine-containing foods (eg, coffee, tea, and chocolate) during past month in the daily diet? | 1. Infrequent ( <3 times per week) 2. Frequent (≥ 3 times per week) |  |

**Schedule E: Dietary diversity**

**5^th^**

**3^rd^**

**2^nd^**

**4^th^**

**1^st^**

Days:

Please describe everything you ate or drank yesterday, including all meals, snacks, and drinks, whether at home or elsewhere. Don't forget to include anything eaten while preparing meals or for others. Let's begin with the first food or drink you had yesterday.

Did you have anything to eat or drink when you woke? If yes, what? Anything else?*

Did you have anything to eat or drink later in the morning? If yes, what? Anything else?*

Did you eat or drink anything at mid-day? If yes, what? Anything else?*

Did you have anything to eat or drink during the afternoon? If yes, what? Anything else?*

Did you have anything to eat in the evening? If yes, what? Anything else?*

Did you have anything else to eat or drink in the evening before going to bed or during the night? If yes, what? Anything else?*

* For each eating episode, ask if the respondent ate or drank anything else until she says "no, nothing else." If a mixed dish like soup or stew is mentioned, ask for all ingredients. For dishes where only broth may be consumed, ask if she had the broth or the ingredients, and continue probing until she says "nothing else."

| Sl  No. | Food Categories | Food items consumed during last 24 hours | Coding category  0= No  1=Yes |
| --- | --- | --- | --- |
| 1. | Food made from grains | Rice, corn/maize, wheat, sorghum, millet or any other grains or foods made from these (e.g. bread, noodles, porridge or other grain products- parata, cha-pati, ruti etc.) |  |
| 2. | White roots and tubers and plantains | White potatoes, taro, white yam, mati alu or any other foods made from white fleshed roots and tubers, or plantains |  |
| 3. | Pulses (beans, peas and lentils) | Mature beans or peas (fresh or dried seed), lentils or bean/pea products |  |
| 4. | Nuts and seeds | Any tree nut, groundnut/peanut or certain seeds, or nut/seed “butters” or pastes |  |
| 5. | Milk and milk products | Milk, cheese, yoghurt or other milk products but not including butter, ice cream, cream or sour cream |  |
| 6. | Organ meat | Liver, kidney, heart or other organ meats or blood-based foods |  |
| 7. | Meat and poultry | Beef, pork, lamb, goat, rabbit, wild game meat, chicken, duck or other birds |  |
| 8. | Fish and seafood | Fresh or dried fish, shellfish or seafood |  |
| 9. | Eggs | Eggs from poultry or any other bird |  |
| 10. | Dark green leafy vegetables | Dark green leafy vegetables, including wild forms + locally available vitamin A rich leaves such as, kalmi, dheki, spinach etc. |  |
| 11. | Vitamin A-rich vegetables, roots and tubers | Pumpkin, carrots, squash or sweet potatoes that are yellow or orange inside + other locally available vitamin A rich vegetables (e.g. red sweet pepper) |  |
| 12. | Vitamin A rich fruits | Ripe mango, ripe papaya, palm , and 100% fruit juice made from these + other locally available vitamin A rich fruits (orange inside) |  |
| 13. | Other vegetables | Other vegetables (e.g. tomato, onion, eggplant) + other locally available vegetables |  |
| 14. | Other fruits | Other fruits, including wild fruits and 100% fruit juice made from these |  |

*** This MDD-W table is designed according to Minimum Dietary Diversity for Women: A Guide to Measurement by FAO of United Nations, 2016.*
