# Supplementary material for: Association between attendance at a behavioral change communication module and dysmenorrhea prevalence among female university students: A propensity score matched comparative study
Source: PLoS One. 2026 May 12;21(5):e0349064. doi: 10.1371/journal.pone.0349064 (PMC13166925; doi:10.1371/journal.pone.0349064)
Supplement: S1 Data — S2 Appendix. Logic model of the BCC module guided by Transtheoretical model (stage of change). S1 File. Informed consent form (ICF). S2 File. Questionnaire in English version. S3 File. Database. S1A Table. Covariate balance before and after propensity score matching under alternative pre-specified model specification (means, %bias, percentage bias reduction, t-test and variance ratios). S1B Table. Overall balance statistics (Rubin’s B and Rubin’s R) under pre-specified propensity score specifications. S2 Table. Adjusted associations of BCC module exposure and key lifestyle factors with dysmenorrhea before and after propensity score matching. S3 Table. Sensitivity analysis: Ordered logistic regression assessing associations of BCC exposure and covariates with four-grade dysmenorrhea severity (unmatched sample, N = 472). S4 Table. Sensitivity analysis of dysmenorrhea prevalence differences under alternative propensity score matching algorithms and specifications. S5 Table. Sensitivity analysis: Adjusted differences in dysmenorrhea prevalence across multiple analytic approaches (ATT and ATE estimates). S6 Table. Sensitivity analysis: Bayesian logistic regression analysis for dysmenorrhea comparing models with and without BCC module exposure. S7 Table. Sensitivity analysis: Corrected adjusted odds ratios (ORs) for the BCC exposure under assumed levels of contamination among non-exposed participants. S1 Fig. Original pamphlet for behavioral change communication (BCC) module. S2 Fig. Distribution of BCC-exposed and non-exposed (control) observations according to whether they are “on support” or “off support” after matching. S1 Text. Calculation of the sample size and proportional distribution among the universities. S2 Text. Explanation of the outcome variable. S3 Text. Detailed information of each covariate. S4 Text. Estimation of BCC associated differences (ATT and ATE estimates) using propensity score matching. S5 Text. Detail calculation of the Log Bayes Factor (LBF). [file pone.0349064.s001.zip › supporting materials/S2 Text.docx]

**S2 Text. Explanation of the outcome variable**

Dysmenorrhea was defined as any pain or discomfort associated with the menstrual cycle. Its severity was assessed using a verbal multidimensional scoring system that categorized dysmenorrhea into four grades based on the intensity of pain, the impact on daily activities, and the need for analgesics. Grade 0, labeled as no pain, referred to the absence of menstrual pain, with no interruption of daily activities or need for analgesics. Grade 1, classified as mild pain, involved slight discomfort that minimally affected daily activities and rarely required analgesics. Grade 2, identified as moderate pain, and described a condition where daily activities were moderately disrupted, necessitating the use of analgesics for relief, although absence from work or school was uncommon. Grade 3, considered severe pain, referred to intense discomfort that significantly hindered daily activities, showed poor response to analgesics, and was often accompanied by vegetative symptoms such as headache, fatigue, nausea, vomiting, and diarrhea [[1](#_ENREF_1)]. Finally, dysmenorrhea pain was classified as a binary outcome variable to align with the assumptions of propensity score analysis. Respondents who reported no pain (graded as 0) were categorized as "No = 0." In contrast, respondents who experienced any level of pain, including Grade 1 (mild), Grade 2 (moderate), or Grade 3 (severe), were combined into a single group and classified as "Yes = 1" for the purposes of this study [[2](#_ENREF_2), [3](#_ENREF_3)].

**Reference**

1. Andersch B and Milsom I. An epidemiologic study of young women with dysmenorrhea*.* Am J Obstet Gynecol. 1982; **144**(6):655-60. https://doi.org/10.1016/0002-9378(82)90433-1 PMID: PMID

2. Austin PC and Stuart EA. Estimating the effect of treatment on binary outcomes using full matching on the propensity score*.* Statistical methods in medical research. 2017; **26**(6):2505-2525 PMID: PMID

3. Yu Y, Zhang M, Shi X, Caram ME, Little RJ, and Mukherjee B. A comparison of parametric propensity score‐based methods for causal inference with multiple treatments and a binary outcome*.* Statistics in Medicine. 2021; **40**(7):1653-1677. https://doi.org/10.1002/sim.8862 PMID: PMID
