# Supplementary material for: Association between attendance at a behavioral change communication module and dysmenorrhea prevalence among female university students: A propensity score matched comparative study
Source: PLoS One. 2026 May 12;21(5):e0349064. doi: 10.1371/journal.pone.0349064 (PMC13166925; doi:10.1371/journal.pone.0349064)
Supplement: S1 Data — S2 Appendix. Logic model of the BCC module guided by Transtheoretical model (stage of change). S1 File. Informed consent form (ICF). S2 File. Questionnaire in English version. S3 File. Database. S1A Table. Covariate balance before and after propensity score matching under alternative pre-specified model specification (means, %bias, percentage bias reduction, t-test and variance ratios). S1B Table. Overall balance statistics (Rubin’s B and Rubin’s R) under pre-specified propensity score specifications. S2 Table. Adjusted associations of BCC module exposure and key lifestyle factors with dysmenorrhea before and after propensity score matching. S3 Table. Sensitivity analysis: Ordered logistic regression assessing associations of BCC exposure and covariates with four-grade dysmenorrhea severity (unmatched sample, N = 472). S4 Table. Sensitivity analysis of dysmenorrhea prevalence differences under alternative propensity score matching algorithms and specifications. S5 Table. Sensitivity analysis: Adjusted differences in dysmenorrhea prevalence across multiple analytic approaches (ATT and ATE estimates). S6 Table. Sensitivity analysis: Bayesian logistic regression analysis for dysmenorrhea comparing models with and without BCC module exposure. S7 Table. Sensitivity analysis: Corrected adjusted odds ratios (ORs) for the BCC exposure under assumed levels of contamination among non-exposed participants. S1 Fig. Original pamphlet for behavioral change communication (BCC) module. S2 Fig. Distribution of BCC-exposed and non-exposed (control) observations according to whether they are “on support” or “off support” after matching. S1 Text. Calculation of the sample size and proportional distribution among the universities. S2 Text. Explanation of the outcome variable. S3 Text. Detailed information of each covariate. S4 Text. Estimation of BCC associated differences (ATT and ATE estimates) using propensity score matching. S5 Text. Detail calculation of the Log Bayes Factor (LBF). [file pone.0349064.s001.zip › supporting materials/S3 Table.docx]

| **Predictable Variables** | **Before propensity score matching**  **N = 472 (non-exposed = 238 and BCC-exposed = 234)** | |
| --- | --- | --- |
|  | **AOR (95% CI)** | **SE** |
| ***Attended BCC module*** |  |  |
| No (ref.) | – |  |
| Yes | 0.33 (0.20, 0.53)*** | 0.08 |
| ***Physical activity*** |  |  |
| Sedentary (ref.) | – |  |
| Active and Athlete | 0.35 (0.20, 0.61)*** | 0.10 |
| ***BMI (Kg/m^2^)*** |  |  |
| Normal weight ( 18.5 – 22.9) (ref.) | – |  |
| Underweight ( < 18.5) | 1.02 (0.59–1.78) | 0.29 |
| Overweight/obese (> 22.9) | 0.75 (0.45–1.24) | 0.19 |
| ***Dietary diversity score (DDS)*** |  |  |
| < 5 (Low) (ref.) | – |  |
| ≥ 5 (High) | 0.34 (0.22–0.54)*** | 0.08 |
| ***Food craving (high fat and sweet food)*** |  |  |
| No (ref.) | – |  |
| Yes | 1.53 (0.99–2.38) | 0.34 |
| ***Skipping breakfast*** |  |  |
| No (ref.) | – |  |
| Yes | 0.95 (0.62–1.46) | 0.21 |
| ***Sleep duration (hours)*** |  |  |
| ≥ 7 hours per night (ref.) | – |  |
| < 7 hours per night | 1.60 (1.03–2.47)* | 0.35 |
| ***Caffeine consumption*** |  |  |
| Infrequent (< 3 times per week) (ref.) | – |  |
| Frequent (≥ 3 times per week) | 1.78 (1.13–2.78)* | 0.41 |
| ***Family history of menstrual disorders*** |  |  |
| No (ref.) | – |  |
| Yes | 1.69 (1.07–2.67)* | 0.39 |
| ***Age at menarche (years)*** |  |  |
| Mean (SD) | 0.73 (0.63–0.85)*** | 0.05 |
| ***Marital status*** |  |  |
| Never Married (ref.) | – |  |
| Ever married | 0.71 (0.33–1.55) | 0.28 |
| ***Father’s educational status*** |  |  |
| Secondary/Higher (> 5 y schooling) (ref.) | – |  |
| Below secondary (0 – 5 y schooling) | 1.06 (0.49–2.28) | 0.41 |
| ***Mother’s educational status*** |  |  |
| Secondary/Higher (> 5 y schooling) (ref.) | – |  |
| Below secondary (0 – 5 y schooling) | 0.62 (0.32–1.21) | 0.21 |
| ***Mother’s occupational status*** |  |  |
| Formal occupation (ref.) | – |  |
| Informal occupation | 0.85 (0.54–1.34) | 0.20 |

**S3 Table. Sensitivity analysis: Ordered logistic regression assessing associations of BCC exposure and covariates with four-grade dysmenorrhea severity (unmatched sample, N = 472)**

*AOR = adjusted odds ratio; SE = standard error; ref. = reference category. Statistical significance is indicated as *p < 0.05, **p < 0.01, and ***p < 0.001. This model is an* ***ordered logistic regression*** *conducted as a sensitivity analysis using the original four-grade dysmenorrhea scale (No pain, mild, moderate, severe). All covariates included in the primary multivariable model were adjusted in this analysis. Estimates reflect* ***associations only*** *and do* ***not imply causality****.*
