# Supplementary material for: Association between attendance at a behavioral change communication module and dysmenorrhea prevalence among female university students: A propensity score matched comparative study
Source: PLoS One. 2026 May 12;21(5):e0349064. doi: 10.1371/journal.pone.0349064 (PMC13166925; doi:10.1371/journal.pone.0349064)
Supplement: S1 Data — S2 Appendix. Logic model of the BCC module guided by Transtheoretical model (stage of change). S1 File. Informed consent form (ICF). S2 File. Questionnaire in English version. S3 File. Database. S1A Table. Covariate balance before and after propensity score matching under alternative pre-specified model specification (means, %bias, percentage bias reduction, t-test and variance ratios). S1B Table. Overall balance statistics (Rubin’s B and Rubin’s R) under pre-specified propensity score specifications. S2 Table. Adjusted associations of BCC module exposure and key lifestyle factors with dysmenorrhea before and after propensity score matching. S3 Table. Sensitivity analysis: Ordered logistic regression assessing associations of BCC exposure and covariates with four-grade dysmenorrhea severity (unmatched sample, N = 472). S4 Table. Sensitivity analysis of dysmenorrhea prevalence differences under alternative propensity score matching algorithms and specifications. S5 Table. Sensitivity analysis: Adjusted differences in dysmenorrhea prevalence across multiple analytic approaches (ATT and ATE estimates). S6 Table. Sensitivity analysis: Bayesian logistic regression analysis for dysmenorrhea comparing models with and without BCC module exposure. S7 Table. Sensitivity analysis: Corrected adjusted odds ratios (ORs) for the BCC exposure under assumed levels of contamination among non-exposed participants. S1 Fig. Original pamphlet for behavioral change communication (BCC) module. S2 Fig. Distribution of BCC-exposed and non-exposed (control) observations according to whether they are “on support” or “off support” after matching. S1 Text. Calculation of the sample size and proportional distribution among the universities. S2 Text. Explanation of the outcome variable. S3 Text. Detailed information of each covariate. S4 Text. Estimation of BCC associated differences (ATT and ATE estimates) using propensity score matching. S5 Text. Detail calculation of the Log Bayes Factor (LBF). [file pone.0349064.s001.zip › supporting materials/S3 text.docx]

**S3 Text. Detailed information of each covariate**

According to the World Health Organization's (WHO) recommendations on physical activity, respondents were categorized as follows: (i) sedentary (referring light-intensity physical activity e.g., casual walking, light household chores; and activities with little or no exercise); (ii) active, (moderate-intensity activities such as brisk walking, light jogging, or moderate exercise e.g. yoga, weight lifting etc. for at least 150–300 minutes per week); and (iii) athlete (vigorous-intensity activities running, fast cycling, jumping, climbing, swimming or competitive sports etc. for at least 75-150 minutes per week) [[1](#_ENREF_1)]. Body Mass Index (BMI, kg/m²) was used as a nutritional indicator to assess obesity. The BMI categories followed the cut-off points recommended by the WHO for the Asian population, which are as follows: (i) underweight (<18.5), (ii) normal weight (18.5 - 22.9), (iii) overweight (23.0 - 27.5), and (iv) obese (>27.5). These thresholds are tailored for the Asian population due to different health risks associated with BMI compared to other populations [[2](#_ENREF_2)]. However, the overweight and obese groups were combined to create an overweight/obese category to avoid problems associated with zero cell counts in estimating the models.

Dietary data were collected using a 24-hour recall method over five consecutive days, and the dietary diversity score (DDS) was calculated following the guidelines from the Food and Agriculture Organization (FAO) [[3](#_ENREF_3)]. Respondents were asked to recall all food items and liquids consumed in the previous 24 hours. A food diary was used to record the dietary intake of respondents for five consecutive days. The consumed food items were grouped into 10 categories: (1) starchy staples including grains, white roots, tubers, and plantains; (2) pulses, such as beans, peas, and lentils; (3) nuts and seeds; (4) dairy products; (5) meat, poultry, and fish; (6) eggs; (7) dark green leafy vegetables; (8) vitamin A-rich fruits and vegetables; (9) other vegetables; and (10) other fruits.

***Table A. Model questionnaire for minimum dietary diversity (MDD) for women***

| **Serial** | **Food Categories** | **Food items** | **If No= 0**  **and**  **If yes=1** |
| --- | --- | --- | --- |
| **A** | Foods made from grains | Rice, corn/maize, wheat, sorghum, millet or any other grains or foods made from these (e.g. bread, noodles, porridge or other grain products- parata, cha-pati, ruti etc.) |  |
| **B** | White roots and tubers and plantains | White potatoes, white yam, mati alu or other foods made from roots |  |
| **C** | Pulses (beans, peas and lentils) | Mature beans or peas (fresh or dried seed), lentils or bean/pea products |  |
| **D** | Nuts and seeds | Any tree nut, groundnut/peanut or certain seeds, or nut/seed “butters” or pastes |  |
| **E** | Milk and milk products |  |  |
| **F** | Organ meat | Liver, kidney, heart or other organ meats or blood-based foods, including from wild game |  |
| **G** | Meat and poultry | Beef, pork, lamb, goat, rabbit, wild game meat, chicken, duck or other bird |  |
| **H** | Fish and seafood | Fresh or dried fish, shellfish or seafood |  |
| **I** | Eggs | Eggs from poultry or any other bird |  |
| **J** | Dark green leafy vegetables | Dark green leafy vegetables, including wild forms and locally available vitamin A rich leaves such as taro leaves, cucumber leaves , bottle guard leaves, spinach etc. |  |
| **K** | Vitamin A-rich vegetables, roots and tubers | Pumpkin, carrot, squash, or sweet potato that are yellow or orange inside and other locally available vitamin A rich vegetables (e.g. red sweet pepper) |  |
| **L** | Vitamin A-rich fruits | Ripe mango, jackfruit, ripe papaya, doub palm (tal palm), wood apple and 100% fruit juice made from these as well as other locally available vitamin A rich fruits (orange inside) |  |
| **M** | Other vegetables | Other vegetables (e.g. tomato, onion, eggplant, ladies finger ) and other locally available vegetables such as bottle guard, bitter guard, bitter guard, cabbage etc. |  |
| **N** | Other fruits | Guava, banana, hog plum, lemon, melon, water melon, litchi, pineapple, jujube, rose apple, elephant apple and custard apple etc. |  |

Data collectors visited respondents every evening for five days to document the dietary recall for the past 24 hours in the diary. The data collection period included one weekend day and four weekdays to capture a representative dietary pattern. For each food group, respondents received a score of 1 if they consumed at least one item from the above food groups in the previous 24 hours or a score of 0 if they did not. However, if the answer of question is coded as "1" for "yes" under either food category “A” or “B,” the respondent is awarded a point for the first food group (“Grains, white roots and tubers, and plantains”). However, no additional point is given if the respondent consumes food items from both categories. To calculate the Dietary Diversity Score (DDS), the scores for the 10 food groups are summed, resulting in a range from 0 to 10. The total DDS for each respondent was calculated for the last 24 hours (Day 1). Similarly, the total DDS was calculated for subsequent days (Day 2, Day 3, Day 4, and Day 5).

***Table B. Aggregation to construct Minimum Dietary Diversity for Women (MDD-W) ****

| **Food categories on model questionnaire** | | **Food groups in (MDD-W)** | |
| --- | --- | --- | --- |
| **Serial** | **Food categories** | **Groups** | **Rename of the food groups for analysis** |
| A | Foods made from grains | 1 | Grains, white roots and tubers, and plantains |
| B | White roots and tubers and plantains |  |  |
| C | Pulses (beans, peas and lentils) | 2 | Pulses (beans, peas and lentils) |
| D | Nuts and seeds | 3 | Nuts and seeds |
| E | Milk and milk products | 4 | Dairy |
| F | Organ meat | 5 | Meat, poultry and fish |
| G | Meat and poultry |  |  |
| H | Fish and seafood |  |  |
| I | Eggs | 6 | Eggs |
| J | Dark green leafy vegetables | 7 | Dark green leafy vegetables |
| K | Vitamin A-rich vegetables, roots and tubers | 8 | Vitamin A-rich fruits and vegetables |
| L | Vitamin A-rich fruits |  |  |
| M | Other vegetables | 9 | Other vegetables |
| N | Other fruits | 10 | Other fruits |

**To construct the MDD-W indicator, the first step is to combine (aggregate) questionnaire rows (food categories those were shown in Table A) into the 10 MDD-W food groups. In the results, at Table 1, this MDD-W was presented as dietary diversity score (DDS).*

The grand total DDS for each participant was then determined by summing the DDS for all five days. To standardize the measure, the **average DDS** was calculated by dividing the grand total DDS by the number of days (5), yielding a value that falls within the range of 0 to 10. Each respondent was categorized as having “High” dietary diversity for an average DDS score of ≥5 or “Low” dietary diversity for a score of <5. The proportion of respondents scoring between 5 and 10 was then calculated to assess overall dietary diversity.

Grand total of DDS = Total DDS (Day 1) + Total DDS (Day 2) + Total DDS (Day 3) + Total DDS (Day 4) + Total DDS (Day 5)

Average DDS $= \frac{Grand total DDS (0- 50)}{Numbers of days (5)}$

Moreover, considering as an important life style factor, the food craving (high fat and sweet food) is defined as a strong urge to consume specific foods including sugary snacks, such as sweets, candy and chocolate; fast food items like burgers, fries, and pizza; and processed foods high in refined carbohydrates, such as pastries and instant noodles or other items, along with any changes in usual eating patterns [[4](#_ENREF_4), [5](#_ENREF_5)]. We extracted data on other various life factors including skipping breakfast one or more times in the last week (yes/no), sleep duration (<7 hours per night; ≥ 7 hours per night), caffeine consumption (infrequent, <3 times per week; frequent, ≥3 times per week) and bedtime habit (23:00 and before; after 23:00) [[6](#_ENREF_6)].

Additionally, socio-demographic and nutritional variables were included as covariates to explore their associations with the outcomes of interest. These covariates comprised the **family history of menstrual disorders** (yes/no), **age at menarche** (in years), **marital status** (never married; ever married), and place of residence (at student dormitory; with family). Father’s and mother’s educational levels were categorized as **below secondary education** (0–5 years of schooling) and **secondary or higher education** (>5 years of schooling). The **mother’s occupation** was classified into two distinct groups: **formal occupations**, which included government jobs, private sector jobs, and retired individuals; and **informal occupations**, encompassing entrepreneurs, fishermen, farmers, and homemakers.

**References**

1. Organization WH. WHO guidelines on physical activity and sedentary behaviour. 2020: World Health Organization.

2. WHO. Appropriate body-mass index for Asian populations and its implications for policy and intervention strategies*.* Lancet. 2004; **363**(9403):157-63. 10.1016/s0140-6736(03)15268-3 PMID: PMID

3. FAO and 360 F. Minimum Dietary Diversity for Women: A Guide for Measurement. 2016, Rome, FAO.

4. Matsuura Y, Inoue A, Kidani M, and Yasui T. Change in appetite and food craving during menstrual cycle in young students*.* Int J Nutr Metab. 2020; **12**(2):25-30. https://doi.org/10.5897/IJNAM2019.0264 PMID: PMID

5. Güzeldere HKB, Efendioğlu EH, Mutlu S, Esen HN, Karaca GN, and Çağırdar B. The relationship between dietary habits and menstruation problems in women: a cross-sectional study*.* BMC Women's Health. 2024; **24**(1):397. 10.1186/s12905-024-03235-4 PMID: PMID

6. Mitsuhashi R, Sawai A, Kiyohara K, Shiraki H, and Nakata Y. Factors associated with the prevalence and severity of menstrual-related symptoms: A systematic review and meta-analysis*.* International Journal of Environmental Research and Public Health. 2022; **20**(1):569 PMID: PMID
