# Supplementary material for: Association between attendance at a behavioral change communication module and dysmenorrhea prevalence among female university students: A propensity score matched comparative study
Source: PLoS One. 2026 May 12;21(5):e0349064. doi: 10.1371/journal.pone.0349064 (PMC13166925; doi:10.1371/journal.pone.0349064)
Supplement: S1 Data — S2 Appendix. Logic model of the BCC module guided by Transtheoretical model (stage of change). S1 File. Informed consent form (ICF). S2 File. Questionnaire in English version. S3 File. Database. S1A Table. Covariate balance before and after propensity score matching under alternative pre-specified model specification (means, %bias, percentage bias reduction, t-test and variance ratios). S1B Table. Overall balance statistics (Rubin’s B and Rubin’s R) under pre-specified propensity score specifications. S2 Table. Adjusted associations of BCC module exposure and key lifestyle factors with dysmenorrhea before and after propensity score matching. S3 Table. Sensitivity analysis: Ordered logistic regression assessing associations of BCC exposure and covariates with four-grade dysmenorrhea severity (unmatched sample, N = 472). S4 Table. Sensitivity analysis of dysmenorrhea prevalence differences under alternative propensity score matching algorithms and specifications. S5 Table. Sensitivity analysis: Adjusted differences in dysmenorrhea prevalence across multiple analytic approaches (ATT and ATE estimates). S6 Table. Sensitivity analysis: Bayesian logistic regression analysis for dysmenorrhea comparing models with and without BCC module exposure. S7 Table. Sensitivity analysis: Corrected adjusted odds ratios (ORs) for the BCC exposure under assumed levels of contamination among non-exposed participants. S1 Fig. Original pamphlet for behavioral change communication (BCC) module. S2 Fig. Distribution of BCC-exposed and non-exposed (control) observations according to whether they are “on support” or “off support” after matching. S1 Text. Calculation of the sample size and proportional distribution among the universities. S2 Text. Explanation of the outcome variable. S3 Text. Detailed information of each covariate. S4 Text. Estimation of BCC associated differences (ATT and ATE estimates) using propensity score matching. S5 Text. Detail calculation of the Log Bayes Factor (LBF). [file pone.0349064.s001.zip › supporting materials/S4 Table.docx]

**S4 Table. Sensitivity analysis of dysmenorrhea prevalence differences under alternative propensity score matching algorithms and specifications**

| **Matching Algorithm** | **Matching Details** | **Matched BCC-exposed (n)** | **Matched non-exposed (Control) (n)** | **ATT (95% CI)** | **Bootstrap SE** |
| --- | --- | --- | --- | --- | --- |
| Nearest neighbor | 1:1, no replacement, caliper 0.01 | 98 | 98 | −0.23 (−0.36, −0.11)*** | 0.06 |
| Nearest neighbor | 1:2, common support | 183 | 121 | −0.25 (−0.41, −0.08)** | 0.08 |
| Nearest neighbor | 1:3, common support | 183 | 150 | −0.27 (−0.43, −0.12)*** | 0.08 |
| Radius matching | Caliper 0.01, common support | 176 | 202 | −0.25 (−0.40, −0.11)** | 0.08 |
| Radius matching | Caliper 0.05, common support | 183 | 238 | −0.27 (−0.40, −0.13)*** | 0.07 |
| Radius matching | Caliper 0.10, common support | 183 | 238 | −0.31 (−0.44, −0.18)*** | 0.07 |
| Kernel matching | Common support | 183 | 234 | −0.27 (−0.40, −0.13)*** | 0.07 |

*ATT = Average difference in dysmenorrhea prevalence among BCC-exposed versus non-exposed participants; SE = Standard error. The main (primary) analysis was conducted using 1:1 nearest neighbor matching (no replacement, caliper 0.01). Propensity scores were estimated using a logistic regression model with the same set of baseline covariates applied across all matching algorithms, based on the best-fitting PSM model. These covariates included physical activity, BMI, dietary diversity, sleep duration, caffeine intake, breakfast skipping, bedtime, family history of menstrual disorders, age at menarche, residence, marital status, and parental education and occupation. Sensitivity analyses were performed using alternative matching algorithms, including nearest neighbor matching (1:2 and 1:3), radius matching with calipers of 0.01, 0.05, and 0.10, and kernel matching. All matching procedures were restricted to the common support region. Standard errors were estimated using 500 bootstrap replications to account for uncertainty in the estimated propensity scores. The consistency of ATT estimates across all matching methods indicates that the association between participation in BCC module and dysmenorrhea prevalence is robust to the choice of matching algorithm and model specification. Statistical significance is identified as * p < 0.05, ** p < 0.01and *** p < 0.001.*
