# Supplementary material for: Association between attendance at a behavioral change communication module and dysmenorrhea prevalence among female university students: A propensity score matched comparative study
Source: PLoS One. 2026 May 12;21(5):e0349064. doi: 10.1371/journal.pone.0349064 (PMC13166925; doi:10.1371/journal.pone.0349064)
Supplement: S1 Data — S2 Appendix. Logic model of the BCC module guided by Transtheoretical model (stage of change). S1 File. Informed consent form (ICF). S2 File. Questionnaire in English version. S3 File. Database. S1A Table. Covariate balance before and after propensity score matching under alternative pre-specified model specification (means, %bias, percentage bias reduction, t-test and variance ratios). S1B Table. Overall balance statistics (Rubin’s B and Rubin’s R) under pre-specified propensity score specifications. S2 Table. Adjusted associations of BCC module exposure and key lifestyle factors with dysmenorrhea before and after propensity score matching. S3 Table. Sensitivity analysis: Ordered logistic regression assessing associations of BCC exposure and covariates with four-grade dysmenorrhea severity (unmatched sample, N = 472). S4 Table. Sensitivity analysis of dysmenorrhea prevalence differences under alternative propensity score matching algorithms and specifications. S5 Table. Sensitivity analysis: Adjusted differences in dysmenorrhea prevalence across multiple analytic approaches (ATT and ATE estimates). S6 Table. Sensitivity analysis: Bayesian logistic regression analysis for dysmenorrhea comparing models with and without BCC module exposure. S7 Table. Sensitivity analysis: Corrected adjusted odds ratios (ORs) for the BCC exposure under assumed levels of contamination among non-exposed participants. S1 Fig. Original pamphlet for behavioral change communication (BCC) module. S2 Fig. Distribution of BCC-exposed and non-exposed (control) observations according to whether they are “on support” or “off support” after matching. S1 Text. Calculation of the sample size and proportional distribution among the universities. S2 Text. Explanation of the outcome variable. S3 Text. Detailed information of each covariate. S4 Text. Estimation of BCC associated differences (ATT and ATE estimates) using propensity score matching. S5 Text. Detail calculation of the Log Bayes Factor (LBF). [file pone.0349064.s001.zip › supporting materials/S4 Text.docx]

**S4 Text. Estimation of BCC associated differences (ATT and ATE estimates) using propensity score matching**

In this study, propensity score methods were applied to estimate differences in dysmenorrhea outcomes associated with participation in the BCC module, while accounting for observed characteristics that differed between participants who exposed to BCC module and those who were not exposed. The analysis focused on two commonly reported estimands in the PSM framework: the average treatment effect on the treated (ATT) and the average treatment effect (ATE).

The ATT represents the average difference in dysmenorrhea outcomes between the observed outcome under BCC exposure and the counterfactual outcome under non-exposure among participants who were exposed to the BCC module. In the potential outcomes framework the ATT is defined as [[1](#_ENREF_1)]:

ATT = E $\left\{ Y\left( 1 \right)-Y(0) | T=1 \right\}$

Where E denotes the expectation among BCC-exposed participants (T = 1); Y(1) represents the observed outcome among BCC-exposed participants; and Y(0) denotes the model-based counterfactual outcome that these same participants would have experienced if they had been non-exposed to the BCC module, given the assumptions of the propensity score model.

Similarly, The ATE reflects the average difference in dysmenorrhea outcomes associated with BCC exposure across the entire study population, comparing outcomes under BCC exposure and non-exposure. It is defined as follows [[1](#_ENREF_1), [2](#_ENREF_2)]:

ATE = E $\left[ Y\left( 1 \right)-Y(0) \right]$

Here E represents the expectation over the entire study population; Y(1) represents the potential outcome under BCC exposure; and Y(0) denotes the corresponding model-based counterfactual outcome under non-exposure to the BCC module.

Both ATT and ATE were estimated using the “teffects” command in Stata 17, adjusting for the same set of covariates included in the optimal propensity score specification. These estimands quantify adjusted differences in dysmenorrhea outcomes between exposure groups under the assumptions of the propensity score framework. However, given the non-randomized cross-sectional design and absence of pre-intervention measurements, the findings should be interpreted as model-based adjusted associations rather than definitive causal effects.

**Reference**

1. Morgan S. Counterfactuals and causal inference. 2015: Cambridge University Press.

2. Imbens GW and Rubin DB. Causal inference in statistics, social, and biomedical sciences. 2015: Cambridge university press.
