# Supplementary material for: Association between attendance at a behavioral change communication module and dysmenorrhea prevalence among female university students: A propensity score matched comparative study
Source: PLoS One. 2026 May 12;21(5):e0349064. doi: 10.1371/journal.pone.0349064 (PMC13166925; doi:10.1371/journal.pone.0349064)
Supplement: S1 Data — S2 Appendix. Logic model of the BCC module guided by Transtheoretical model (stage of change). S1 File. Informed consent form (ICF). S2 File. Questionnaire in English version. S3 File. Database. S1A Table. Covariate balance before and after propensity score matching under alternative pre-specified model specification (means, %bias, percentage bias reduction, t-test and variance ratios). S1B Table. Overall balance statistics (Rubin’s B and Rubin’s R) under pre-specified propensity score specifications. S2 Table. Adjusted associations of BCC module exposure and key lifestyle factors with dysmenorrhea before and after propensity score matching. S3 Table. Sensitivity analysis: Ordered logistic regression assessing associations of BCC exposure and covariates with four-grade dysmenorrhea severity (unmatched sample, N = 472). S4 Table. Sensitivity analysis of dysmenorrhea prevalence differences under alternative propensity score matching algorithms and specifications. S5 Table. Sensitivity analysis: Adjusted differences in dysmenorrhea prevalence across multiple analytic approaches (ATT and ATE estimates). S6 Table. Sensitivity analysis: Bayesian logistic regression analysis for dysmenorrhea comparing models with and without BCC module exposure. S7 Table. Sensitivity analysis: Corrected adjusted odds ratios (ORs) for the BCC exposure under assumed levels of contamination among non-exposed participants. S1 Fig. Original pamphlet for behavioral change communication (BCC) module. S2 Fig. Distribution of BCC-exposed and non-exposed (control) observations according to whether they are “on support” or “off support” after matching. S1 Text. Calculation of the sample size and proportional distribution among the universities. S2 Text. Explanation of the outcome variable. S3 Text. Detailed information of each covariate. S4 Text. Estimation of BCC associated differences (ATT and ATE estimates) using propensity score matching. S5 Text. Detail calculation of the Log Bayes Factor (LBF). [file pone.0349064.s001.zip › supporting materials/S5 Table.docx]

**S5 Table. Sensitivity analysis: Adjusted differences in dysmenorrhea prevalence across multiple analytic approaches (ATT and ATE estimates)**

| **Estimation approach** | **ATT (95% CI)** | **Robust SE (ATT)** | **ATE (95% CI)** | **Robust SE (ATE)** |
| --- | --- | --- | --- | --- |
| Regression adjustment (RA) | −0.23 (−0.33, −0.13)*** | 0.05 | −0.24 (−0.33, −0.14)*** | 0.05 |
| Inverse probability weighting (IPW) | −0.23 (−0.34, −0.12)*** | 0.05 | −0.24 (−0.36, −0.14)*** | 0.05 |
| IPWRA (doubly robust) | −0.23 (−0.33, −0.13)*** | 0.05 | −0.24 (−0.34, −0.14)*** | 0.05 |
| Propensity score matching (neighbor 1) | −0.27 (−0.40, −0.13)*** | 0.07 | −0.23 (−0.35, −0.11)*** | 0.06 |
| Propensity score matching (neighbor 2) | −0.21 (−0.34, −0.08)** | 0.07 | −0.20 (−0.31, −0.08)** | 0.06 |
| Nearest neighbor matching | −0.23 (−0.37, −0.10)** | 0.07 | −0.22 (−0.35, −0.09)** | 0.07 |
| Nearest neighbor matching (bias adjusted) | −0.21 (−0.33, −0.10)*** | 0.06 | −0.23 (−0.34, −0.13)*** | 0.06 |

*Estimates represent adjusted differences in dysmenorrhea prevalence between BCC-exposed and non-exposed participants. ATT (average difference among BCC-exposed participants) is the primary estimand highlighted in the main manuscript, while ATE (average difference in the overall study population) is presented for comparison and robustness. Groups were made comparable using matching-based procedures, and differences were calculated as mean differences in the matched sample. RA = regression adjustment; IPW = inverse probability weighting; IPWRA = inverse probability weighting with regression adjustment (doubly robust estimator). Robust standard errors (SE) are reported for each estimate. Statistical significance is indicated by * p < 0.05, ** p < 0.01, and *** p < 0.001. Covariate balance between the BCC-exposed and non-exposed groups after matching was excellent (P > χ² = 1.000).*
