# Supplementary material for: Association between attendance at a behavioral change communication module and dysmenorrhea prevalence among female university students: A propensity score matched comparative study
Source: PLoS One. 2026 May 12;21(5):e0349064. doi: 10.1371/journal.pone.0349064 (PMC13166925; doi:10.1371/journal.pone.0349064)
Supplement: S1 Data — S2 Appendix. Logic model of the BCC module guided by Transtheoretical model (stage of change). S1 File. Informed consent form (ICF). S2 File. Questionnaire in English version. S3 File. Database. S1A Table. Covariate balance before and after propensity score matching under alternative pre-specified model specification (means, %bias, percentage bias reduction, t-test and variance ratios). S1B Table. Overall balance statistics (Rubin’s B and Rubin’s R) under pre-specified propensity score specifications. S2 Table. Adjusted associations of BCC module exposure and key lifestyle factors with dysmenorrhea before and after propensity score matching. S3 Table. Sensitivity analysis: Ordered logistic regression assessing associations of BCC exposure and covariates with four-grade dysmenorrhea severity (unmatched sample, N = 472). S4 Table. Sensitivity analysis of dysmenorrhea prevalence differences under alternative propensity score matching algorithms and specifications. S5 Table. Sensitivity analysis: Adjusted differences in dysmenorrhea prevalence across multiple analytic approaches (ATT and ATE estimates). S6 Table. Sensitivity analysis: Bayesian logistic regression analysis for dysmenorrhea comparing models with and without BCC module exposure. S7 Table. Sensitivity analysis: Corrected adjusted odds ratios (ORs) for the BCC exposure under assumed levels of contamination among non-exposed participants. S1 Fig. Original pamphlet for behavioral change communication (BCC) module. S2 Fig. Distribution of BCC-exposed and non-exposed (control) observations according to whether they are “on support” or “off support” after matching. S1 Text. Calculation of the sample size and proportional distribution among the universities. S2 Text. Explanation of the outcome variable. S3 Text. Detailed information of each covariate. S4 Text. Estimation of BCC associated differences (ATT and ATE estimates) using propensity score matching. S5 Text. Detail calculation of the Log Bayes Factor (LBF). [file pone.0349064.s001.zip › supporting materials/S5 Text.docx]

**S5 Text. Detail calculation of the Log Bayes Factor (LBF)**

The log LBF was calculated using Bayesian logistic regression models fitted in Stata. Two competing models were specified: a null model excluding the BCC module attendance and an alternative model including the BCC module attendance, while both models adjusted for the same set of covariates. The LBF was calculated according to the following stata command:

bayes, rseed (123456) mcmcsize (40000) burnin (5000) thinning (5): logit var (outcome) var (independent)

bayes, rseed (123456) mcmcsize (40000) burnin (5000) thinning (5): logit var (outcome) var (BCC-exposure) var (independents)

Bayesian estimation was performed using weakly informative normal priors for all regression coefficients (mean = 0, SD = 2.5) and for the intercept (mean = 0, SD = 10), with a Bernoulli likelihood specified for the binary outcome (dysmenorrhea).

Posterior distributions of the model parameters were approximated using Markov Chain Monte Carlo (MCMC) sampling with a Random-walk Metropolis–Hastings algorithm. A total of 40,000 iterations were performed, with the first 5,000 iterations discarded as burn-in to allow for convergence. Thinning was applied by retaining every fifth sample to reduce autocorrelation, resulting in 7,000 posterior samples used to summarize associations between covariates and dysmenorrhea. Posterior means of the coefficients were reported as odds ratios, with 95% credible intervals (CrIs) shown for the strength and direction of these associations [[1](#_ENREF_1), [2](#_ENREF_2)]. All analyses were conducted with a fixed random seed (rseed = 123456) to ensure reproducibility. Separate analyses were performed for unmatched and matched samples to examine the consistency of associations under different covariate balance conditions.

Following model estimation, the log marginal likelihood was obtained for each model. The log marginal likelihood reflects the probability of the observed data under a given model and serves as the basis for Bayesian model comparison [[3](#_ENREF_3)]. The LBF was computed as the difference between the log marginal likelihood of the alternative model and that of the null model:

LBF = Log marginal likelihood (Alternative) − Log marginal likelihood (Null)

Positive LBF values indicate stronger relative support for the model including BCC attendance compared with the model excluding it [[4](#_ENREF_4)]. The LBF was used to assess the relative evidence for including BCC attendance in the model, rather than to establish causal effects.

**Reference**

1. Mira A. *MCMC Methods to Estimate Bayesian Parametric Models*, in *Handbook of Statistics*, D.K. Dey and C.R. Rao, Editors. 2005, Elsevier. p. 415-436.

2. Kim H. Monte Carlo Statistical Methods*.* Technometrics. 2000; **42**(4):430-431. 10.1080/00401706.2000.10485722 PMID: PMID

3. Du H, Edwards MC, and Zhang Z. Bayes factor in one-sample tests of means with a sensitivity analysis: A discussion of separate prior distributions*.* Behav Res Methods. 2019; **51**(5):1998-2021. 10.3758/s13428-019-01262-w PMID: PMID

4. Lodewyckx T, Kim W, Lee MD, Tuerlinckx F, Kuppens P, and Wagenmakers E-J. A tutorial on Bayes factor estimation with the product space method*.* Journal of Mathematical Psychology. 2011; **55**(5):331-347. https://doi.org/10.1016/j.jmp.2011.06.001 PMID: PMID
