# Supplementary material for: Association between attendance at a behavioral change communication module and dysmenorrhea prevalence among female university students: A propensity score matched comparative study
Source: PLoS One. 2026 May 12;21(5):e0349064. doi: 10.1371/journal.pone.0349064 (PMC13166925; doi:10.1371/journal.pone.0349064)
Supplement: S1 Data — S2 Appendix. Logic model of the BCC module guided by Transtheoretical model (stage of change). S1 File. Informed consent form (ICF). S2 File. Questionnaire in English version. S3 File. Database. S1A Table. Covariate balance before and after propensity score matching under alternative pre-specified model specification (means, %bias, percentage bias reduction, t-test and variance ratios). S1B Table. Overall balance statistics (Rubin’s B and Rubin’s R) under pre-specified propensity score specifications. S2 Table. Adjusted associations of BCC module exposure and key lifestyle factors with dysmenorrhea before and after propensity score matching. S3 Table. Sensitivity analysis: Ordered logistic regression assessing associations of BCC exposure and covariates with four-grade dysmenorrhea severity (unmatched sample, N = 472). S4 Table. Sensitivity analysis of dysmenorrhea prevalence differences under alternative propensity score matching algorithms and specifications. S5 Table. Sensitivity analysis: Adjusted differences in dysmenorrhea prevalence across multiple analytic approaches (ATT and ATE estimates). S6 Table. Sensitivity analysis: Bayesian logistic regression analysis for dysmenorrhea comparing models with and without BCC module exposure. S7 Table. Sensitivity analysis: Corrected adjusted odds ratios (ORs) for the BCC exposure under assumed levels of contamination among non-exposed participants. S1 Fig. Original pamphlet for behavioral change communication (BCC) module. S2 Fig. Distribution of BCC-exposed and non-exposed (control) observations according to whether they are “on support” or “off support” after matching. S1 Text. Calculation of the sample size and proportional distribution among the universities. S2 Text. Explanation of the outcome variable. S3 Text. Detailed information of each covariate. S4 Text. Estimation of BCC associated differences (ATT and ATE estimates) using propensity score matching. S5 Text. Detail calculation of the Log Bayes Factor (LBF). [file pone.0349064.s001.zip › supporting materials/S6 table.docx]

**S6 Table. Sensitivity analysis: Bayesian logistic regression analysis for dysmenorrhea comparing models with and without BCC module exposure**

| **Covariates/LBF estimation** | **Unmatched Sample**  **N = 472**  **(non-exposed = 238 and BCC-exposed = 234)** | | **Matched Sample**  **N = 196**  **(non-exposed = 98 and BCC-exposed = 98)** | | **Remarks** |
| --- | --- | --- | --- | --- | --- |
|  | **Null Model**  **Odds ratio (OR)**  **(95% credible intervals)** | **Alternative Model**  **Odds ratio (OR)**  **(95% credible intervals)** | **Null Model**  **Odds ratio (OR)**  **(95% credible intervals)** | **Alternative Model**  **Odds ratio (OR)**  **(95% credible intervals)** |  |
| ***Attended BCC module*** |  |  |  |  |  |
| Yes | — | **0.13 (0.06–0.24)** | — | **0.15 (0.06–0.29)** | Inversely associated with dysmenorrhea prevalence |
| ***Physical activity*** |  |  |  |  |  |
| Active and Athlete | 0.21 (0.11–0.35) | 0.28 (0.14–0.51) | 0.20 (0.07–0.45) | 0.13 (0.06–0.23) | Strong inverse association |
| ***BMI (kg/m^2^)*** |  |  |  |  |  |
| Underweight (< 18.5) | 1.20 (0.53–2.31) | 1.17 (0.56–2.06) | 1.40 (0.48–3.34) | 1.19 (0.47–2.55) | No clear association |
| Overweight/obese (> 22.9) | 2.52 (1.09–4.99) | 2.54 (1.01–5.37) | 2.63 (0.89–6.30) | 3.14 (1.21–6.91) | Positively associated |
| ***Dietary diversity score*** |  |  |  |  |  |
| ≥5 (High) | 0.17 (0.09–0.29) | 0.13 (0.06–0.23) | 0.11 (0.04–0.22) | 0.06 (0.02–0.13) | Strong inverse association |
| ***Food craving (high-fat and sweet foods)*** |  |  |  |  |  |
| Yes | 2.74 (1.45–4.73) | 2.01 (1.03–3.62) | 2.77 (1.15–5.71) | 2.76 (1.08–5.95) | Positively associated |
| ***Skipping breakfast*** |  |  |  |  |  |
| Yes | 1.87 (1.00–3.20) | 1.16 (0.59–2.04) | 0.93 (0.39–1.93) | 1.02 (0.41–2.06) | No clear association |
| ***Sleep duration*** |  |  |  |  |  |
| <7 hours/night | 2.45 (1.34–4.23) | 2.05 (0.99–3.81) | 1.14 (0.47–2.31) | 1.22 (0.53–2.37) | No clear association |
| ***Caffeine consumption*** |  |  |  |  |  |
| Frequent ( ≥ 3 times per week) | 2.36 (1.17–4.30) | 2.00 (1.01–3.46) | 1.64 (0.72–3.25) | 1.32 (0.57–2.65) | Association attenuated after matching |
| ***Family history of menstrual disorders*** |  |  |  |  |  |
| Yes | 1.26 (0.54–2.61) | 1.46 (0.63–3.00) | 1.32 (0.52–2.81) | 1.22 (0.48–2.60) | No clear association |
| ***Age at menarche (years)*** | 0.81 (0.68–0.95) | 0.78 (0.69–0.92) | 1.05 (0.86–1.27) | 0.91 (0.82–1.02) | Association only in unmatched sample |
| ***Marital status*** |  |  |  |  |  |
| Ever married | 1.61 (0.38–4.46) | 1.07 (0.31–2.64) | 1.61 (0.26–5.18) | 2.20 (0.57–5.87) | No clear association |
| ***Father’s educational status*** |  |  |  |  |  |
| Bellow secondary | 4.37 (1.24–11.55) | 3.86 (1.02–10.91) | 1.32 (0.35–3.47) | 2.41 (0.59–6.71) | Association attenuated after matching |
| ***Mother’s educational status*** |  |  |  |  |  |
| Bellow secondary | 0.73 (0.28–1.66) | 0.51 (0.21–1.05) | 1.15 (0.40–2.79) | 0.80 (0.39–1.50) | No clear association |
| ***Mother’s occupational status*** |  |  |  |  |  |
| Informal | 0.69 (0.34–1.26) | 1.07 (0.50–1.99) | 1.06 (0.39–2.25) | 1.13 (0.41–2.49) | No clear association |
| **LBF = LML (alternative) – LML ( null)** | –163.26 – (–175.52) = **12.26** | | –100.01– (–101.01) **= 1.00** | | Very strong evidence (unmatched sample) and weak evidence (matched sample) in favor of the association between BCC exposure and dysmenorrhea prevalence |

*The log Bayes factor = LBF and LML = Log Marginal Likelihood. Posterior means of the coefficients are reported as odds ratios, with 95% credible intervals (CrIs) shown in parentheses. A variable is considered credibly associated with the outcome if its 95% CrI does not include the null value (OR = 1). Bayesian logistic regression models were estimated using a Bernoulli likelihood with a logit link. Weakly informative normal priors were specified for all regression coefficients (mean = 0, SD = 2.5) and for the intercept (mean = 0, SD = 10). MCMC sampling was performed with 40,000 iterations, a burn-in of 5,000 iterations, thinning every 5^th^ sample, and a fixed random seed (rseed = 123456) to ensure reproducibility. The LBF was calculated as the difference between the log marginal likelihood of the alternative model (including the BCC module exposure) and the null model (excluding the BCC module exposure): LBF = LML (Alternative) – LML (Null). Larger positive LBF values indicate stronger evidence that including the BCC module is associated with the outcome.*
