# Supplementary material for: Association between attendance at a behavioral change communication module and dysmenorrhea prevalence among female university students: A propensity score matched comparative study
Source: PLoS One. 2026 May 12;21(5):e0349064. doi: 10.1371/journal.pone.0349064 (PMC13166925; doi:10.1371/journal.pone.0349064)
Supplement: S1 Data — S2 Appendix. Logic model of the BCC module guided by Transtheoretical model (stage of change). S1 File. Informed consent form (ICF). S2 File. Questionnaire in English version. S3 File. Database. S1A Table. Covariate balance before and after propensity score matching under alternative pre-specified model specification (means, %bias, percentage bias reduction, t-test and variance ratios). S1B Table. Overall balance statistics (Rubin’s B and Rubin’s R) under pre-specified propensity score specifications. S2 Table. Adjusted associations of BCC module exposure and key lifestyle factors with dysmenorrhea before and after propensity score matching. S3 Table. Sensitivity analysis: Ordered logistic regression assessing associations of BCC exposure and covariates with four-grade dysmenorrhea severity (unmatched sample, N = 472). S4 Table. Sensitivity analysis of dysmenorrhea prevalence differences under alternative propensity score matching algorithms and specifications. S5 Table. Sensitivity analysis: Adjusted differences in dysmenorrhea prevalence across multiple analytic approaches (ATT and ATE estimates). S6 Table. Sensitivity analysis: Bayesian logistic regression analysis for dysmenorrhea comparing models with and without BCC module exposure. S7 Table. Sensitivity analysis: Corrected adjusted odds ratios (ORs) for the BCC exposure under assumed levels of contamination among non-exposed participants. S1 Fig. Original pamphlet for behavioral change communication (BCC) module. S2 Fig. Distribution of BCC-exposed and non-exposed (control) observations according to whether they are “on support” or “off support” after matching. S1 Text. Calculation of the sample size and proportional distribution among the universities. S2 Text. Explanation of the outcome variable. S3 Text. Detailed information of each covariate. S4 Text. Estimation of BCC associated differences (ATT and ATE estimates) using propensity score matching. S5 Text. Detail calculation of the Log Bayes Factor (LBF). [file pone.0349064.s001.zip › supporting materials/S6 Text.docx]

**S6 Text. Sensitivity Analysis for Potential Contamination**

To address the potential risk of contamination between the BCC-exposed and non-exposed participants—since both groups were recruited from the same universities and shared dormitory environments—a sensitivity analysis was conducted. Contamination was defined as the indirect exposure of control participants to BCC module content through peer interactions.

The primary multivariable logistic regression model (**S2 Table**) estimated the association between attendance at the BCC module and dysmenorrhea, adjusting for covariates included in the optimal propensity score model: physical activity, BMI, dietary diversity score, food cravings, breakfast skipping, sleep duration, caffeine consumption, family history of menstrual disorders, age at menarche, marital status, and parental education and occupation. To evaluate the potential impact of contamination, corrected odds ratios (ORs) for BCC attendance were estimated under different assumed contamination levels using the following formula:

$${OR}_{corrected}= {OR}_{observed}^{1/(1-c)}$$

where ${OR}_{observed}$ is the adjusted OR for BCC attendance obtained from multivariable logistic regression, and “c” represents the proportion of control participants assumed to be indirectly exposed to the BCC module (assumed 10%, 20%, 30%, 40%, 50%). This analysis evaluates how different degrees of contamination might influence the magnitude of the observed association. The corrected ORs are scenario-based estimates and do not have confidence intervals, as they are deterministic transformations of the observed OR.

***Stata commands Used***

*Step 1: Run multivariable logistic regression*

logit (dysmenorrhea) (attendance in BCC module) (covariates)

*Step 2: Store observed OR for BCC intervention*

scalar OR_obs = exp(_b[1.BCC attendance])

display "Observed OR = " OR_obs

*Step 3: Correct OR for different hypothetical contamination levels*

scalar OR_c10 = OR_obs^(1/(1-0.10))

display "Corrected OR (10% contamination) = " OR_c10

scalar OR_c20 = OR_obs^(1/(1-0.20))

display "Corrected OR (20% contamination) = " OR_c20

scalar OR_c30 = OR_obs^(1/(1-0.30))

display "Corrected OR (30% contamination) = " OR_c30

scalar OR_c40 = OR_obs^(1/(1-0.40))

display "Corrected OR (40% contamination) = " OR_c40

scalar OR_c50 = OR_obs^(1/(1-0.50))

display "Corrected OR (50% contamination) = " OR_c50
