# Supplementary material for: Association between attendance at a behavioral change communication module and dysmenorrhea prevalence among female university students: A propensity score matched comparative study
Source: PLoS One. 2026 May 12;21(5):e0349064. doi: 10.1371/journal.pone.0349064 (PMC13166925; doi:10.1371/journal.pone.0349064)
Supplement: S1 Data — S2 Appendix. Logic model of the BCC module guided by Transtheoretical model (stage of change). S1 File. Informed consent form (ICF). S2 File. Questionnaire in English version. S3 File. Database. S1A Table. Covariate balance before and after propensity score matching under alternative pre-specified model specification (means, %bias, percentage bias reduction, t-test and variance ratios). S1B Table. Overall balance statistics (Rubin’s B and Rubin’s R) under pre-specified propensity score specifications. S2 Table. Adjusted associations of BCC module exposure and key lifestyle factors with dysmenorrhea before and after propensity score matching. S3 Table. Sensitivity analysis: Ordered logistic regression assessing associations of BCC exposure and covariates with four-grade dysmenorrhea severity (unmatched sample, N = 472). S4 Table. Sensitivity analysis of dysmenorrhea prevalence differences under alternative propensity score matching algorithms and specifications. S5 Table. Sensitivity analysis: Adjusted differences in dysmenorrhea prevalence across multiple analytic approaches (ATT and ATE estimates). S6 Table. Sensitivity analysis: Bayesian logistic regression analysis for dysmenorrhea comparing models with and without BCC module exposure. S7 Table. Sensitivity analysis: Corrected adjusted odds ratios (ORs) for the BCC exposure under assumed levels of contamination among non-exposed participants. S1 Fig. Original pamphlet for behavioral change communication (BCC) module. S2 Fig. Distribution of BCC-exposed and non-exposed (control) observations according to whether they are “on support” or “off support” after matching. S1 Text. Calculation of the sample size and proportional distribution among the universities. S2 Text. Explanation of the outcome variable. S3 Text. Detailed information of each covariate. S4 Text. Estimation of BCC associated differences (ATT and ATE estimates) using propensity score matching. S5 Text. Detail calculation of the Log Bayes Factor (LBF). [file pone.0349064.s001.zip › supporting materials/S7 Table.docx]

**S7 Table. Sensitivity analysis: Corrected adjusted odds ratios (ORs) for the BCC exposure under assumed levels of contamination among non-exposed participants**

| **Assumed Contamination in non-exposed (control) Group** | **Corrected Adjusted OR** | **Interpretation** |
| --- | --- | --- |
| 0% (observed) | 0.13 | Observed OR without assumed contamination |
| 10% | 0.10 | Slight indirect exposure; the association is somewhat stronger than observed, suggesting minimal dilution. |
| 20% | 0.08 | Moderate contamination; the estimated association strengthens further, indicating that partial exposure among controls would attenuate the observed association. |
| 30% | 0.05 | High contamination; the association remains inverse and meaningful, showing that indirect exposure is unlikely to fully explain the observed differences. |
| 40% | 0.03 | Very high contamination; the inverse association persists, suggesting robustness of the observed pattern despite substantial potential contamination. |
| 50% | 0.02 | Extreme contamination; the association remains strongly inverse, indicating that indirect exposure among non-exposed participants alone cannot account for the observed differences. |

*Corrected adjusted ORs were estimated under varying hypothetical levels of contamination among non-exposed participants. Contamination refers to potential indirect exposure to BCC module content. Across all assumed scenarios, the direction of the association between BCC module exposure and dysmenorrhea remained inverse, with corrected ORs below 1.0.*
